# Nerve growth factor activates autophagy in Schwann cells to enhance myelin debris clearance and to expedite nerve regeneration

**DOI:** 10.7150/thno.40919

**Published:** 2020-01-01

**Authors:** Rui Li, Duohui Li, Chengbiao Wu, Libing Ye, Yanqing Wu, Yuan Yuan, Shengnan Yang, Ling Xie, Yuqin Mao, Ting Jiang, Yiyang Li, Jian Wang, Hongyu Zhang, Xiaokun Li, Jian Xiao

**Affiliations:** 1 Department of Hand Surgery and Peripheral Neurosurgery, The First Affiliated Hospital and School of Pharmaceutical Sciences, Wenzhou Medical University, Wenzhou, Zhejiang, 325000, China; 2 Research Center, Affiliated Xiangshang Hospital, Wenzhou Medical University, Ningbo, Zhejiang, 315700, China; 3 PCFM Lab, School of Chemistry, Sun Yat-sen University, Guangzhou, Guangdong 510127, China; 4 The Institute of Life Sciences, Wenzhou University, Wenzhou 325035, China

**Keywords:** Myelin debris clearance, Autophagic flux, Nerve growth factor (NGF), Schwann cells, Nerve regeneration

## Abstract

**Rationale**: Autophagy in Schwann cells (SCs) is crucial for myelin debris degradation and clearance following peripheral nerve injury (PNI). Nerve growth factor (NGF) plays an important role in reconstructing peripheral nerve fibers and promoting axonal regeneration. However, it remains unclear if NGF effect in enhancing nerve regeneration is mediated through autophagic clearance of myelin debris in SCs.

**Methods**: *In vivo*, free NGF solution plus with/without pharmacological inhibitors were administered to a rat sciatic nerve crush injury model. *In vitro*, the primary Schwann cells (SCs) and its cell line were cultured in normal medium containing NGF, their capable of swallowing or clearing degenerated myelin was evaluated through supplement of homogenized myelin fractions.

**Results**: Administration of exogenous NGF could activate autophagy in dedifferentiated SCs, accelerate myelin debris clearance and phagocytosis, as well as promote axon and myelin regeneration at early stage of PNI. These NGF effects were effectively blocked by autophagy inhibitors. In addition, inhibition of the p75 kD neurotrophin receptor (p75^NTR^) signal or inactivation of the AMP-activated protein kinase (AMPK) also inhibited the NGF effect as well.

**Conclusions**: NGF effect on promoting early nerve regeneration is closely associated with its accelerating autophagic clearance of myelin debris in SCs, which probably regulated by the p75^NTR^/AMPK/mTOR axis. Our studies thus provide strong support that NGF may serve as a powerful pharmacological therapy for peripheral nerve injuries.

## Introduction

Traumatic peripheral nerve damage is a chronic disease resulting in significant disability 1. Establishing an optimal microenvironment that favors axonal regrowth and remyelination is vital for peripheral nerve regeneration and functional recovery 2, 3. Following axonal trauma, the distal fibers are disconnected from the neuronal stump and undergo Wallerian degeneration (WD), in which the neural cytoskeleton becomes disintegrated to produce a large quantity of axonal and myelin debris 4, 5. In addition, the presence of myelin sheet fragments surrounding the lesion sites further compounds the difficulty of nerve regeneration. Therefore, the rate and extent of myelin debris clearance is essential for effective nerve repair after injury 6.

Schwann cells (SCs) in the peripheral nervous system (PNS), play an active role in removing myelin debris 7, 8. Upon axonal injury, SCs begin to dedifferentiate and undergo extensive proliferation to acquire increased intrinsic digestive capacity, which affords SCs the ability to remove myelin fragments, often with the cooperation of macrophages 9. For instance, during WD, SCs interacted with macrophages to catabolize myelin sheath segments into smaller intracellular debris 10, 11. Subsequently, SCs migrate to form bands of Büngner that guide the regenerative axon to the target organs via a direct pathway. Meanwhile, the newly synthesized axonal and myelin proteins largely occurred at as early as 14 days post-injury 1, 12. Therefore, targeting SCs to eliminate myelin debris will be a powerful means for axon regeneration and reinnervation. However, the precise molecular mechanisms by which SCs mediate myelin fragment clearance are yet to be defined.

Macroautophagy (hereafter called autophagy) is an intracellular dynamic process for degrading senescent or damaged organelles/proteins 13, through which damaged organelles and pathological proteins are encapsulated in autolysosomes and are subsequently degraded by the lysosome. Conversely, dysregulation of autophagy is linked to a growing number of diseases, including neurodegenerative diseases, infections, and inflammation 14, 15. In neurons, autophagy has a protective effect that is essential for neuronal maintenance and survival 16. Autophagy thus offers an important cellular protection mechanism against various pathological processes 17, 18.

The role of autophagy in SCs in peripheral nerve injury (PNI) and repair has been investigated. SC autophagy has been found to be beneficial for scar reduction and myelination 19, which plays an important role in preventing or delaying the onset and chronification of neuropathic pain and neuropathy 20, 21. Moreover, recent evidence has revealed that SCs initiate myelin breakdown via selective autophagy during WD 22, 23. Rapamycin, an activator of autophagy, is effective in promoting nerve regeneration and motor recovery in a sciatic nerve crush model 24. Additionally, autophagy is involved in structural plasticity during the myelination process 25. For instance, conditional knockouts of the ATG-7 gene, an important autophagy regulator, resulted in accumulation of cytosolic organelles and proteins during segmental demyelination, as a result, causing a delay in myelin clearance 26. Therefore, enhancing the autophagy pathway at the early phase after PNI is a new therapeutic strategy for peripheral nerve repair.

Nerve growth factor (NGF), the first isolated neurotrophic factor, has been shown to stimulate neuronal survival, and promote axonal growth and elongation 27-34. Importantly, NGF has a robust neuroprotective effect against nerve system disorders, especially PNI 35, 36. NGF exerts its effects through the two Type-1 cell surface receptors, the 140 kD tyrosine kinase receptor A (TrkA) and the 75 kD neurotrophin receptor (p75^NTR^) 31-34, 37-41. Although p75^NTR^ has been thought to be a death signaling molecule that can initiate cell apoptosis under certain conditions 42, recent data suggest that increasing p75^NTR^ expression also contributes to neuronal survival and regeneration in injured nerves 43-45. SCs also express abundant p75^NTR^ during development 46. NGF is capable of binding to p75^NTR^ with high affinity in SCs following PNI 47. The NGF/ p75^NTR^-medicated signaling pathways in SCs have been linked to cell proliferation, myelination, and synaptic plasticity 48-50.

The AMP-activated protein kinase (AMPK), a cellular energy modulator, is also involved in regulating autophagy. Under stress conditions, such as starvation and debris accumulation, AMPK activates autophagy or enhances autophagic flux by repressing the mammalian target of the rapamycin (mTOR), a negative regulator of autophagy 51, 52.

However, little is known about the role and mechanisms of NGF/p75^NTR^ signaling in SCs in mediating rapid and efficient clearance of myelin debris to shorten the time of peripheral nerve regeneration during WD. In the present study, we investigated the effect and molecular mechanism of NGF on myelin fragment clearance using a sciatic nerve crush injury PNI rat model. We found that NGF administration accelerated the degradation and removal of myelin during WD. NGF treatment significantly shortened the time of regrowth and remyelination in crushed nerves. We further showed that these effects by NGF are likely mediated by the p75^NTR^/AMPK/mTOR dependent pathways to enhance autophagic activities in SCs.

## Materials and methods

### Ethical Statement

All procedures and protocols involving the use and care of animals were approved by the Institutional Animal Care and Use Committee of Wenzhou Medical University according strictly to the National Institutes of Health Guide.

### Reagents and antibodies

We purchased each from the indicated suppliers: NGF (Sigma-Aldrich, SRP3015), Compound C (Cpd C, an AMPK inhibitor, Aladdin, D139352), 3-methyladenine (3-MA, an autophagy inhibitor, Aladdin, M129496), chloroquine (CQ; a lysosomal inhibitor, Aladdin, C193834), TAT-Pep5 (the p75^NTR^ inhibitor, Merck Millipore, 506181), hematoxylin and eosin (HE, Beycotime Biotechnology, C0105), Masson's trichrome staining (Masson, Solarbio, G1340), a luxol fast blue kit (LFB, Solarbio, G3245), toluidine blue solution (TB, Solarbio, G3665), oil red O solution (ORO, Solarbio, G1260), and 4' 6-diamidino-2-phenylindole-dihydrochloride (DAPI, Beycotime Biotechnology, C1006. The following antibodies against proteins were used in the study: anti-*p*-AMPK (CST, #2535), anti-AMPK (CST, #5831), anti-*p*-p70s6k (CST, #9204), anti-p70s6k (CST, #2708), anti-*p*-mTOR (CST, #5536), anti-mTOR (CST, #2083), anti-p75^NTR^ (Abcam, ab52987), anti-beclin-1 (Abcam, ab62557), anti-P62 (Abcam, ab56416), anti-LC3 (Abcam, ab128025), anti-MBP (Abcam, ab62631), anti-MPZ (Abcam, ab31851), anti-S-100 (Abcam, ab4066), anti-GFAP (Abcam, ab10062), NF-200 (Abcam, ab4680), LAMP1 (Abcam, ab25639), β-actin (Abcam, ab8227), anti-ATG7 (Bioworld, BS6046), anti-ATG5 (Bioworld, AP6026), GAPDH (Millipore, AB2302), anti-CD68 (Abcam, ab955).

### Sciatic nerve crush model and drug treatments

Wistar male rats of 8 weeks old were obtained from the Laboratory Animals Center of Wenzhou Medical University. Rats were adapted to the local animal facilities (5 rats per cage) and maintained at controlled temperature (23 ± 2 °C) and relative humidity (50-60%) with free access to water and regular food. All animals were habituated in the animal care facility for ≥7 days before the experiments.

The procedure for the sciatic nerve surgery has been described previously 53. Briefly, rats were anesthetized by i.p. injection of 4% pentobarbital sodium (30 mg/kg) before surgery. The upper thigh was shaved and sterilized using iodophor. The sciatic nerve in the right mid-thigh was exposed and visualized via blunt dissection using glass needle and clamped for 2 min with a pair of vascular clips (approximately 2-mm distance, 30 g force, Oscar, China), The surgical wounds were then closed with 4-0 stitches.

Following surgery, each rat with sciatic nerve crushing rapidly received 0.2 mL NGF solution (20 μg mL^-1^) 54 or an equal volume of saline via intramuscular injection once daily for 5 days. The sciatic nerve crushed rats administered with NGF was as the NGF group or the PNI+NGF group. The remaining injured rats administered with saline treatment were as the PNI group. For the sham operation group, the rats were subjected to exposure of the sciatic nerve without contusion. Each group contained 8 rats. To explore the effect of NGF on AMPK signaling or autophagic activation, the inhibitor Cpd C (20 mg kg^-1^) 55 or 3-MA (50 mg kg^-1^) 24 was administered via intraperitoneal administration at days 1 and 3. Both of these two drugs were firstly dissolved in sterile saline to get the final concentration of 20 mg mL^-1^ (Cpd C) and 50 mg mL^-1^ (3-MA), respectively. To determine NGF signaling via the p75^NTR^, TAT-Pep5 was intravenously injected into each animal at 40 μg 56 provided 4 h prior to the injection of NGF solution and then administered once per day until the rats were sacrificed. This drug was directly solubilized in sterile saline to obtain the final concentration of 0.2 mg mL^-1^ before treatment. To exclude NGF-TrkA signaling, Trk A inhibitor K252a (200 nM, merck, 420298) or GW441756 (10 mg/kg, selleck, S2891) was intraperitoneally injected for continuous 5 days after nerve contusion 57, 58. K252a was initially dissolved in DMSO (10 mM) and then diluted in sterile saline to achieve the working solution with the final concentration of 200 nM. As for GW441756, it was originally dissolved in DMSO (1 g mL^-1^) and then diluted in sterile saline to achieve the working solution with the final concentration of 10 mg mL^-1^. To evaluate autophagic flux, CQ was intraperitoneally (i.p.) administered at the dose of 10 μg kg^-1^ 20 2 h before the animals were sacrificed. The purchased chloroquine (CQ) powder was initially diluted in DMSO (25 mM or 8 mg mL^-1^ stock) and further diluted in the saline to a concentration of 1 μg mL^-1^. All the procedures were going under the dark environment.

### Lentivirus injection

To knockdown AMPK or LC3β expression in SD rats. We carried out orthotopical injection (OI) of lentivirus (LV)-RNAi to the contusive nerve with a Hamilton microsyringe. We also generated a lentiviral vector that expresses scrambled species as a negative control (NC). Control LV-RNAi, LV-AMPK-RNAi/LV-NC_AMPK_-RNAi and LV-LC3β-RNAi/LV-NC_LC3β_-RNAi were all purchased from Shanghai GeneChem Company (Shanghai, China). Following sciatic nerve crush, rats were injected with 2 µL of LV-AMPK-RNAi/LV-NC_AMPK_-RNAi or LV-LC3β-RNAi/LV-NC_LC3β_-RNAi containing 2 × 10^8^ TU mL^-1^. The rats receiving NGF plus LV-NC_AMPK_-RNAi, LV-AMPK-RNAi, LV-NC_LC3β_-RNAi or LV-LC3β-RNAi were designated as: NC-AMPK, LV-AMPK, NC-LC3β or LV-LC3β, respectively. Animals were evaluated blindly with respect to experimental conditions.

### Tissue preparation

**Figure 1:**
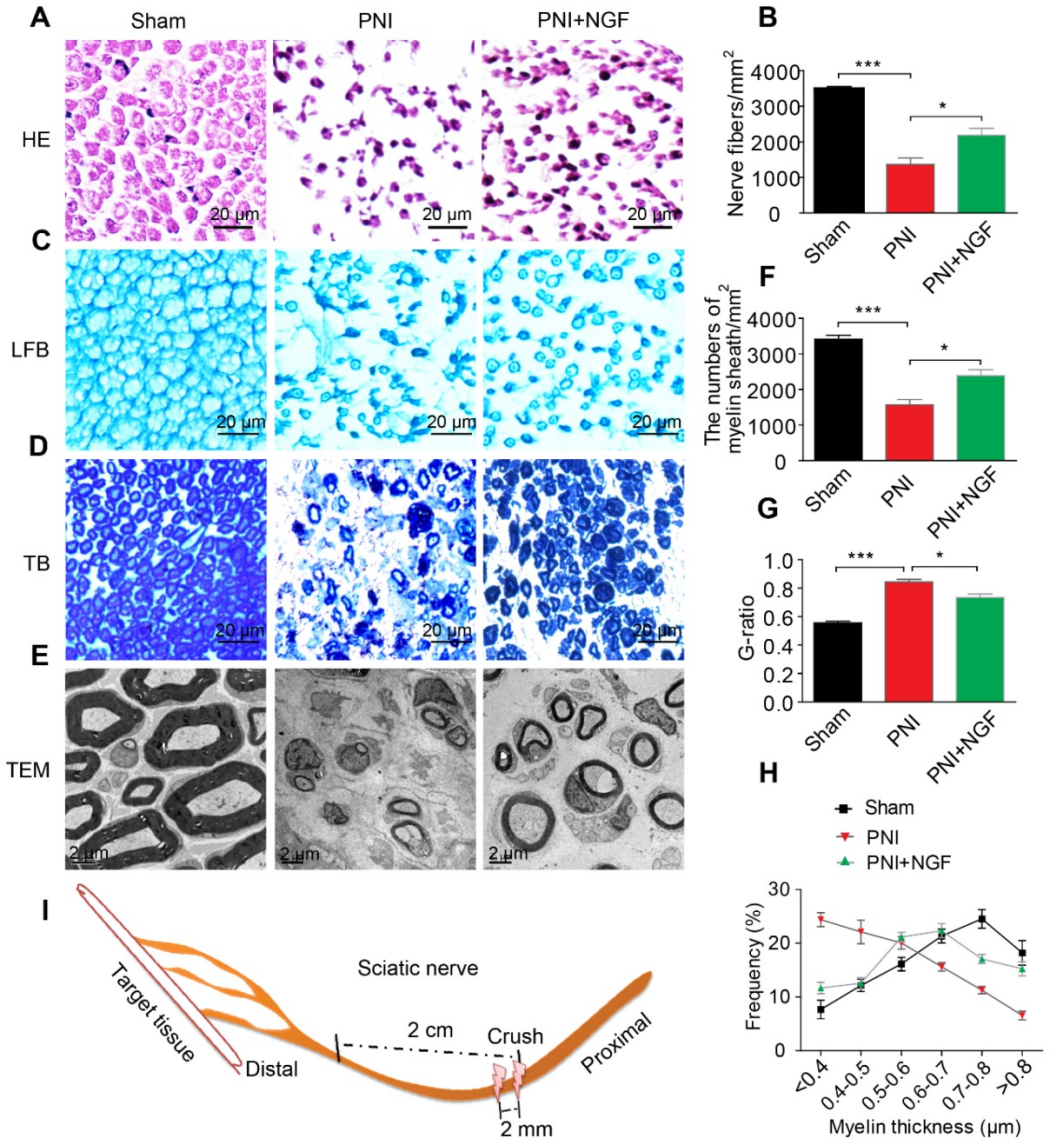
** NGF expedites axon regeneration and remyelination after PNI. (A)** Representative HE staining images of sciatic nerve lesion cross-sections from the sham, PNI model, and PNI+NGF groups at 14 days following injury. **(B)** Statistical analysis of the number of nerve fibers in each group. Data are presented as the mean ± SEM; n = 3 rats per group. Nerve fibers: F_(2, 6)_ = 44.74, ^***^P_sham vs PNI_ < 0.001, ^*^P_PNI vs PNI+NGF_ = 0.042. **(C-E)** Transverse higher-magnification images of sciatic nerves in the three groups. FBL, TB and electron micrographs were also used to evaluate myelin regeneration at 14 days after surgery. Scale bars represent 20 µm (FBL and TB) and 2 µm (TEM). **(F)** The number of myelin sheaths per 1 mm^2^ in the three groups. Data are presented as the mean ± SEM; n = 3 rats per group. F_(2, 6)_ = 41.80, ^***^P_sham vs PNI_ < 0.001, ^*^P_PNI vs PNI+NGF_ = 0.035. **(G)** Quantification of the G-ratio in the three groups. Data are presented as the mean ± SEM; n = 3 rats per group. F_(2, 6)_ = 59.26, ^***^P_sham vs PNI_ < 0.001, ^*^P_PNI vs PNI+NGF_ = 0.037. **(H)** Quantification of the frequency distribution profile of the thickness of myelin sheaths.** (I)** A schematic showing the nerve segments collected in each group.

At 5 or 14 days post nerve crush, animals were anesthetized with 10% chloral hydrate (3.5 mL kg^-1^) and transcardially perfused with 0.1 M phosphate-buffered saline (PBS; pH = 7.35). Degenerated or regenerating nerves were removed and collected in an Eppendorf tube (2 mL) for further experiments. For general staining, a 2-cm length of nerve segment at the lesion site (a schematic diagram of harvested nerve segments is shown in Figure 1I) was fixed in 4% paraformaldehyde overnight. The next day, the tissues were dehydrated with an alcohol gradient, embedded in paraffin. The 2 mm segments within the injuried region was cut into 5-μm-thick transections and mounted on poly-L-lysine-coated slides. For immunofluorescence, the dehydrated sciatic nerve segments of each group soaked in 30% sucrose solution were embedded in optimal cutting temperature compound (OTC) for frozen sections. These longitudinal sections were serially sectioned at 5 μm thickness on a freezing microtome (Leica CM1520, Hesse-Darmstadt, Germany). The capturing images were viewed at the contusion area (2 mm length). For Western blotting and real-time polymerase chain reaction (RT-PCR), the samples (1 cm length) at the contusion area were dissected and immediately stored at -80°C. The diagrammatic sketch of tissue handling and slice observation was shown in Figure S1.

### Red Oil O Staining

The longitudinal frozen sections in different groups were fixed in 4% polyoxymethylene for 20 min. The slides were then dehydrated in 100% propylene glycol for 10 min after washing in PBS for 3 times. The sections were stained with 0.5% Red Oil O solution (Solarbio, G1260) at 25°C for 10 min. Nuclei were stained with hematoxylin (Beycotime Biotechnology, C0105) for 5 min. Finally, the coverslips were mounted (Gel Mount, BioMeda, USA) and examined by a light microscope (Nikon Eclipse 80i, Tokyo, Japan).

### Histological assessments

Slides containing paraffin embedded nerve sections were first dried in an oven and subsequently dewaxed and rehydrated in xylene and ethanol, respectively. The procedures of HE, Masson, LFB, TB staining were performed according to the manufacturer's instructions. ORO staining was performed on sciatic nerve longitudinal frozen sections. Lastly, all dyed simples were imaged with a light microscope (Nikon Eclipse 80i, Tokyo, Japan). The quantified index of nerve fibers/mm^2^ and myelin numbers/mm^2^ were defined as dividing the total nerve fibers (HE) or myelin numbers (TB) by the selected region area. The quantified index of the average collagen area per nerve unit was defined as dividing the integrated option density (IOD) in a visual field by its target area. The images were imported to the Image-Pro Plus software (Media Cybernetics Inc, Silver Spring, MD, USA) to automatically obtain data for the total nerve fibers, myelin number, IOD and corresponding target region areas. We randomly selected three visual fields in each section for recording and analysis. Six sections were selected for each rat. Each group contained 3 animals.

### Electron microscopy

For transmission electron microscopy (TEM), the crush sciatic nerve sites (cutting into 1 mm within the 2 mm crushed region) were fixed overnight by immersion in 2.5% glutaraldehyde. The next day, samples were postfixed with 1% osmium and 1% uranyl acetate for 1 h respectively, dehydrated in ethanol, and embedded in resin. The crushed portion of the samples was cut into ultrathin sections (50 nm thick) to receive staining with uranyl acetate/lead citrate. The quantitative indexes including the number of newborn and abnormal myelin sheathes were measured from three randomly selected fields from one section, and a total of six sections from five samples for each group was used for capturing images with a Hitachi H-600 TEM (HITACH, Tokyo, Japan).

### Immunofluorescence analysis

Standard immunohistochemistry procedures were described previously 53, 59. The samples harvested at 5 days were labeled with the following primary antibodies: anti-S-100 (labeling SCs, 1:1000) and anti-LC3 (labeling autophagy, 1:1000), anti-MPZ (labeling myelin, 1:1000) and anti-GFAP (labeling dedifferentiated SCs, 1:500), anti-GFAP and anti-p75^NTR^ (NGF receptor, 1:1000). The samples collected at 14 days were double-stained with anti-NF-200 (labeling axon, 1:100,000) and anti-MBP (myelin marker, 1:1000). The sections were sequentially incubated with FITC-conjugated anti-rabbit IgG (Abcam, ab150073) or TRITC-conjugated anti-mouse IgG (Abcam, ab7065). The statistical indicators of the immunofluorescence staining were measured in three randomly selected fields from one section, and a total of six sections from three animals of each group was used for capturing images with a Nikon confocal laser microscope (Nikon, A1 PLUS, Tokyo, Japan) or a Nikon Eclipse 80i fluorescence microscope. For different antibody immunoreactivity positive areas (%), the calculation formula used was IOD / selected region area*100%. The IOD and selected region area were measured using the Image pro-plus software.

### Western blotting analysis

The lesioned sciatic nerve tissues were lysed in a Laemmli sample buffer (2% SDS, 52.5 mM Tris-HCl PH 6.8, and protein inhibitors). The concentration of protein lysates was determined using the Micro BCA Protein Assay Kit (Beycotime Biotechnology, P0010). Eighty micrograms of proteins were separated by SDS-PAGE and transferred onto PVDF membranes (Millipore, Bedford, MA). Afterwards, the membranes were blocked with 5% nonfat milk and were probed with primary antibodies including: ATG-7 (1:1000), ATG-5 (1:1000), P62 (1:1000), Beclin-1 (1:1000), LC3 (1:1000), *p*-AMPK (1:500), AMPK (1:1000), *p*-p70s6k (1:500), p70s6k (1:1000), *p*-mTOR (1:500), mTOR (1:1000), MBP (1:1000), and MPZ (1:1000) overnight at 4°C. The next day, the membranes were incubated with horseradish peroxidase-conjugated secondary antibodies (1:10,000; Bioworld; anti-rabbit, BS13278; anti-mouse, BS50350) for 1 h. Immunoreactive bands were visualized using the ChemiDic TM XRS + Imaging System (Bio-Rad, 1708195). Densitometric quantification of the membranes was obtained using Image J software (National Institutes of Health, USA). Experiments were repeated three times and GAPDH (1:10,000) or β-actin (1:5000) was used as an internal control.

### Quantitation of RT-PCR

The total RNA of the thawed nerve tissue samples was extracted using Trizol reagent and reverse-transcribed into complementary DNA (cDNA) using the Prime Script™ RT reagent Kit (TaKaRa, Japan, RR047Q) according to manufacturer's instruction. Quantitative expression of myelination and function-associated genes, including MBP, MPZ, MAG, GAP43 and MAP-2, was run in parallel with each primer set in RT-PCR with bio-radiQ™ SYBR® Green Supermix (Bio-Rad, USA, #170-882AP). β-Actin served as an internal control. The forward and reverse primer sequences are shown in Table 1. Data analyses were performed using the SDS Enterprise Database software according to previous reports 60. All experiments were performed in triplicate.

### Schwann cell phagocytosis assay in vitro

Myelin debris were collected from uncut sciatic nerve extracts in adult SD rats by density gradient centrifugation, based on a modified version of the procedure by Larocca and Norton reports 61, 62. Briefly, sciatic nerves were exposed, then removed to a culture dish filled with calcium/magnesium-free Hank's buffered solution to carefully strip off the connective tissues surrounding the nerves under a stereomicroscope. The sciatic nerves were collected into a 1.5 ml Eppendorf tube and suspended in 0.27 M sucrose solution containing 20 mM Tris-Cl buffer at pH 7.45, followed by homogenization with a homogenizer (PRO 200, USA). The homogenized myelin fractions were supplemented into medium containing RSC 96 cells (a rat Schwann cell line, purchased from ScienCell Research Laboratories), which were seeded into 6-well plates with a density of 5 × 10^6^ cells/well. After culturing for 24 h, myelin debris were added to the medium for culturing another 24 h (recorded as 0 h). Then, the medium was supplemented with/without NGF solution at doses of 50 ng mL^-1 63^ and cultured for another 12 h, 24 h or 48 h. The RSC 96 cells harvested at different time points were co-immunolabeling for MBP and S-100. We regarded the cell medium containing only myelin debris as the control group. The cell medium with myelin debris and NGF added was taken as the NGF group.

To quantify MBP immunoreactivity, 6 randomly photomicrographs per group at each timepoint were captured. This experiment was performed in triplicate. Then, these acquired images were imported into Image-Pro Plus software and analyzed to achieve the integrated option density (IOD) and corresponding target distribution area. The IOD value was divided by corresponding target distribution area to obtain mean density (MD). Next, The MBP immunoreactivity (%) at different times in each group was calculated as following equation: The MBP immunoreactivity (%) = (MD_different time points_-MD_begin_) / (MD_0h_-MD_begin_) × 100%.

### Myelin phagocytosis assays using primary Schwann cells

An established protocol was followed 64. Briefly, primary SCs (Cat# EM1010, Boston, MA, USA) were cultured in 6-well plates at the density of 1.00×10^6^ cells/well. The next day, cells in each well were rinsed once with phosphate buffer saline (PBS) and incubated with 1mL complete media supplemented with 10% Fetal Bovine Serum (FBS) and 800 μg mL^-1^ pHrodo™ Red, succinimidyl ester (pHrodo, Thermo Fisher Scientific, P36600)-labeled myelin debris for 24 h. The procedures of peripheral nerve system (PNS) myelin purification and conjugation with pHrodo were referred to the previous description 65, 66. The SC media was immediately added the NGF solution at doses of 50 ng mL^-1^ 63 after mixing with pHrodo-conjugated myelin debris. Then, the SCs were cultured in a humidified incubator (37 °C, 5% CO_2_). 6 h later, the experimental medium was supplemented with 5 mM 3-MA 67 (Aladdin, M129496). Then, the primary SCs were cultured for another 18 h. The SC media containing with/without NGF was named as control and NGF groups, respectively. If the media also added with 3-MA after culturing for 6 h, we regarded this group as NGF+3-MA.

To reveal engulfed pHrodo-conjugated particles, live SCs in each well were imaged at a 1 h interval for 24 h using a Nikon ECLIPSE Ti microscope (Nikon, Japan). For image processing analysis, we took 5 images/well using 10× objective lens from random areas of the 6 well plates and calculated the integrated fluorescence intensity using the Image J software.

### Detection of autophagic flow

RSC 96 Schwann cell lines were seeded on 6-well plates and transfected with tandem fluorescent mRFP-GFP-LC3 adenoviral vectors (HanBio, Shanghai, China) when the confluence reached to 50-70%. After 24 h transfection, the culture medium was changed back to complete medium. Meanwhile, the medium was added in 100 µM H_2_O_2_ plus with/without NGF (50 ng/mL). Afterwards, cells were incubated in this condition for 4 h again at 37°C. Cellular autophagosomes and autolysosomes were detected by a Nikon Eclipse 80i fluorescence microscope. GFP degrades in acidic environment while mRFP does not. This is because the pKa value of GFP was relatively higher than mRFP (pKa_GFP_ = 6.0, pKam_RFP_ = 4.5). Therefore, yellow spots (i.e., RFP^+^GFP^+^) indicate autophagosomes, while only red puncta indicate autophagolysosomes (i.e., RFP^+^GFP^-^). The numbers of yellow and red-only puncta in the merged image were analyzed by Image-Pro Plus software according to the manufacturer's manual. Representative images of confocal microscopy from three independent experiments performed in duplicates. Six different fields were randomly taken for each sample in one experiment.

### Data presentation and statistical analysis

To determine statistical significance, the differences between two groups were analyzed by the unpaired Student's t-test with Welch's correction. For three groups or more, one-way ANOVA with a Bonferroni post hoc comparison was used. For one-way ANOVA statistical evaluations, F values were presented in the format F_(degree of freedom 1, degree of freedom 2)_ = X. The degrees of freedom were computed as degree of freedom 1= k - 1, in which k was the number of compared treatments, and degree of freedom 2= n - k in which n was the total number of samples across the treatment groups. For Student's t-test, the values of t and d.f. were showed as the format t = X', d.f. = n' - 2, in which n' was the total number of samples in two compared groups. All parametric data were obtained using GraphPad Prism 5 Software. (GraphPad Software Inc., La Jolla, CA, USA), and *P*-values < 0.05 were considered statistically significant. Graph bars indicate the mean and standard error of the mean (SEM) in all results.

## Results

### NGF promotes nerve repair at the early stage of nerve regeneration

NGF plays key roles in neuroprotection and neurogenesis 68-70. To examine whether NGF had an effect during the early stage of nerve injury, we injected NGF or vehicle control for 5 consecutive days in situ at the right hindlimb of each animal using a rat sciatic nerve crush PNI model. 14 days post-operation, the status of nerve regeneration was measured by hematoxylin and eosin (HE). As shown in Figure 1A- B, we observed an increase in the number of regenerated nerve fibers in the PNI+NGF group relative to the PNI control group.

**Figure 2:**
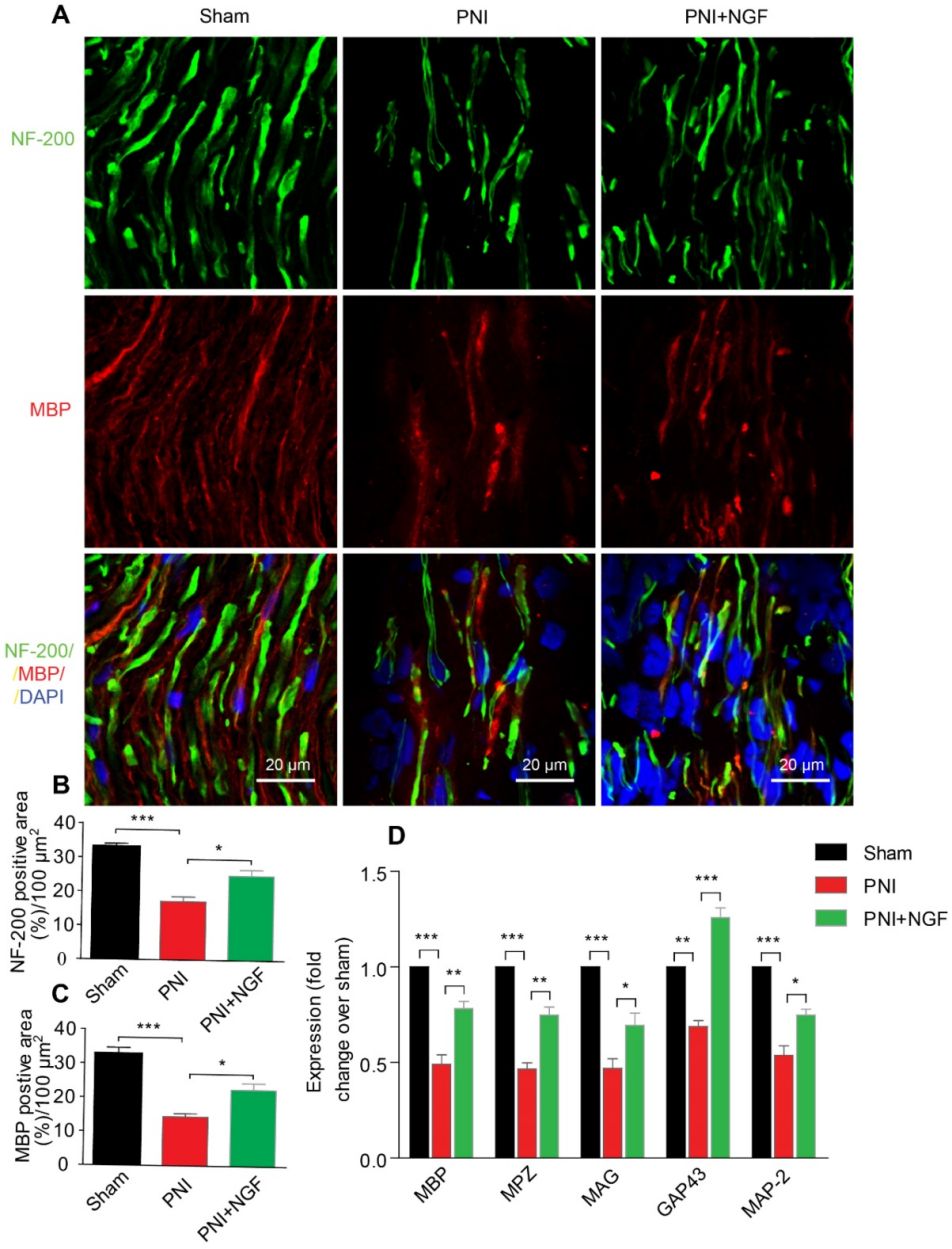
** NGF enhances neuronal regrowth following PNI. (A)** A representative micrograph of NF-200 (green) and MBP (red) immunofluorescence in each group. DAPI: nuclear staining (blue). **(B, C)** Quantification of NF-200 and MBP-positive areas per 100 µm^2^ in each group. Data are presented as the mean ± SEM; n = 4 rats per group. MBP: F_(2, 9)_ = 29.77, ^***^P_sham vs PNI_ < 0.001, ^*^P_PNI vs PNI+NGF_ = 0.019; NF-200: F_(2, 9)_ = 31.01, ^***^P_sham vs PNI_ < 0.001, ^*^P_PNI vs PNI+NGF_ = 0.018. **(D)** RT-PCR analysis of the expression of myelinated and functional response genes in the lesion nerve treated with/without NGF at 14 days post-injury. Data are presented as the mean ± SEM. n = 3 independent experiments. MBP F_(2, 6)_ = 49.51, ^***^P_sham vs PNI_ < 0.001, ^**^P_PNI vs PNI+NGF_ = 0.0091; MPZ F_(2, 6)_ = 76.08,^ ***^P_sham vs PNI_ < 0.001, ^**^P_PNI vs PNI+NGF_ = 0.0062; MAG F_(2, 6)_ = 33.03, ^***^P_sham vs PNI_ < 0.001, ^*^P_PNI vs PNI+NGF_ = 0.048; GAP43 F_(2, 6)_ = 69.15, ^**^P_sham vs PNI_ = 0.0043,^ ***^P_PNI vs PNI+NGF_ < 0.001; MAP-2: F_(2, 6)_ = 46.07, ^***^P_sham vs PNI_ < 0.001, ^*^P_PNI vs PNI+NGF_ = 0.024.

Next, we defined the effect of NGF on remyelination through Luxol fast blue (FBL) staining, toluidine blue (TB) staining and transmission electron microscopy (TEM) (Figure 1C-E). Consistent with the results of morphological analysis, regenerative myelin sheaths in the PNI control group were thinner and smaller than those in the NGF treatment group, which was reflected in the number of regenerated myelin (Figure 1F), the G-ratio measurement (Figure 1G) and the myelin thickness analysis. The ranking order of myelin thickness (from thick to thin) is the sham group-the PNI+NGF group-the PNI control group (Figure 1H). In addition, immunofluorescence double-staining results for MBP (a major constituent of the myelin sheath) and NF-200 (neurofilament, marker for axons) also showed a significant higher immunoreactivity of MBP and NF-200 in the PNI+NGF group, compared to that in the PNI control group (Figure 2A-C). In addition, exogenous NGF administration significantly upregulated the expression of myelination and function-associated genes including MBP, MPZ, MAG, GAP43 and MAP-2 (Figure 2D). These results demonstrated that NGF indeed promoted axon regeneration and remyelination at the early stage of injury.

We next investigated if NGF effect on neurons contributed to axon regeneration and remyelination at the early stage of injury**.** NGF is known to exert its biological functions through activating two different surface receptors, the 140 kD tyrosine receptor kinase A (TrkA) and the 75 kD neurotrophic factor receptor (p75^NTR^). While SCs express predominantly p75^NTR^, peripheral sensory neurons such as dorsal root ganglion (DRG) have both TrkA and p75^NTR^ 71. We first confirmed that NGF signaled through TrkA to promote axonal growth in DRG. We extracted and cultured DRGs from 2 month-old adult rats in neural medium supplemented with 50 ng/mL NGF 72. We used GW441756 (final concentration: 2 nM) to inhibit TrkA mediated signaling 73 and TAT-Pep5 (final concentration: 10 µM) to suppress the p75^NTR^ function 74. After 3 days of treatments, we measured the length and density of axon using immunostaining. Adult rat DRGs cultured in NGF alone exhibited dense axonal growth with extensive elongation. Treatment with GW441756, but not with TAT-Pep5, inhibited NGF-induced axonal growth (Figure S2). These findings have established that NGF acts on TrkA, but not p75^NTR^, in neurons to promote axonal growth and extension.

To further establish if or not NGF effected on neurons to contribute to myelin removal and nerve fiber regeneration, we tested the in vivo effect of two TrkA inhibitors (K252a, GW441756) to block NGF/TrkA signaling in neurons. Following PNI, rats were treated with either NGF alone or with NGF+K252a (200 nM/1 mL/per animal) 58 once a day for 5 days. 14 days after PNI, we measured the extent of nerve recovery using both H&E and double staining for NF-200/MBP. As illustrated in Figure S3A- B (Left panel) and Figure S3C-E, neither the numbers of nerve fibers nor the immunoreactivity of NF-200/MBP showed a significant difference between the NGF group and the NGF+ K252a group. Similar results were also achieved with the use of another TrkA inhibitor GW441756 (Figure S4K and Figure S4M-P). In contrast, suppressing p75^NTR^ activation with TAT-Pep5 delayed axonal regrowth and remyelination (Figure S3A-B Right panel and Figure S3C-E). Therefore, inhibiting NGF/TrkA in neurons had no appreciable effect in NGF-mediated nerve recovery under our experimental settings. Taken together, our results have suggested that NGF acts on p75^NTR^ to promote nerve regeneration at early stage of PNI.

### NGF accelerates SC-mediated myelin debris clearance

**Figure 3:**
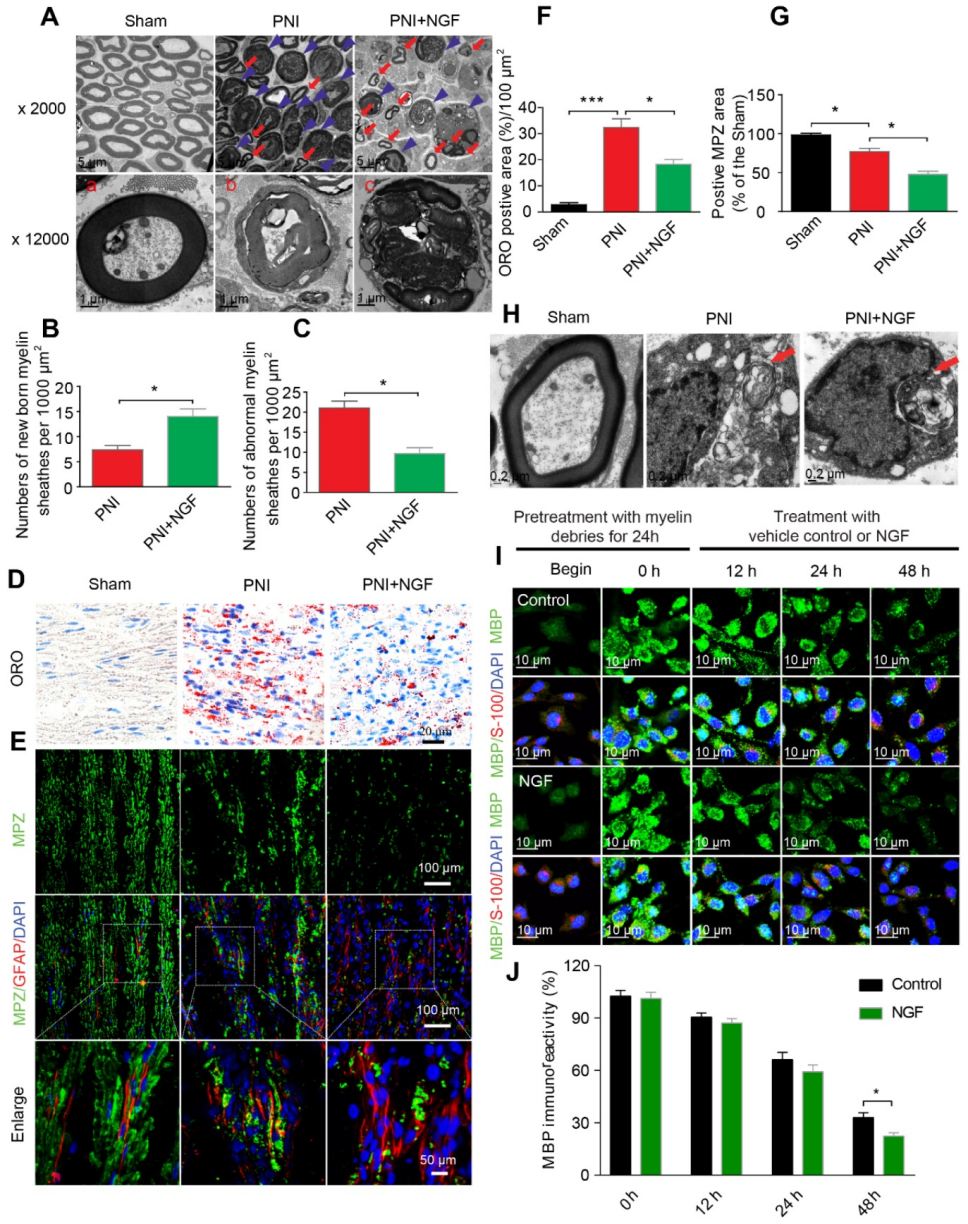
** NGF accelerates SC-dependent myelin degradation. (A)** Electron micrographs of sciatic nerve cross sections at day 5 post-injury in the sham, PNI and PNI+NGF groups. The images represent morphological profiles of myelin in each group. The magnified images below show single typical normal myelin (a), demyelinated or degenerative myelin (b), and disintegrating myelin (c). The abnormal myelin includes b and c. **(B, C)** Quantification of the numbers of newborn and abnormal myelin sheaths per 1000 μm^2^ in the PNI and PNI+NGF groups. Data are presented as the mean ± SEM; n = 5 rats per group. Newborn myelin: ^*^P_PNI vs PNI+NGF_ = 0.019, t = 3.780, d.f. = 8; Abnormal myelin:^ *^P_PNI vs PNI+NGF_ = 0.015, t = 5.013, d.f. = 8. **(D)** Staining of myelin debris with ORO was performed on sciatic nerve longitudinal sections from the three groups at 5 days post-injury. **(E)** Immunofluorescence images showing MPZ (green) and GFAP (red) in sciatic nerve sections taken from each group at 5 days post-injury. Nuclei were labeled with DAPI (blue).** (F)** Quantitative results showing the ORO-positive area per 100 μm^2^ from (D). Data are the mean values ± SEM; n = 3 rats per group. F_(2, 6)_ = 50.66, ^***^P_sham vs PNI_ < 0.001, ^*^P_PNI vs PNI+NGF_ = 0.021. **(G)** Quantification of MPZ-positive area (%) in each sciatic nerve tissue sample. Data are the mean values ± SEM; n = 3 rats per group. F_(2, 6)_ = 48.35, ^*^P_sham versus PNI_ = 0.020, ^*^P_PNI vs PNI+NGF_ = 0.015.** (H)** Electron micrograph showing the presence of a fragmented myelin (red arrows)-containing Schwann cell cytoplasmic pocket in the PNI and PNI+NGF groups and a normal myelin in the sham group.** (I)** Double**-**immunostaining showing MPZ^+^ myelin inclusions and S-100 markers for SCs in normal cultured RSC96 cells (begin). 24 h after incubation with myelin debris, 50 ng/mL NGF was added to the culture medium (0 h) and incubated for 12 h, 24 h and 48 h, respectively.** (J)** Quantitation of MBP immunoreactivity (relative to 0 h) at different time points in group control and NGF. Data are the mean values ± SEM; n = 3 independent experiments with a minimum of 6 picture frames analyzed per time point/group/experiment. T = 48 h ^*^P_NGF versus control_ = 0.036, t = 3.113, d.f. = 4.

Degradation of myelin debris by SCs during the first 5-7 days after injury is a prerequisite for nerve regeneration 75. We wondered if NGF-mediated neural regeneration was correlated with the speed of myelin clearance following nerve injury. To address this issue, we first prepared distal segments of each right sciatic nerve for morphometric studies using TEM (Figure 3A). In sham rats, the myelin sheaths were compactly arranged and clearly visible. After sciatic nerve injury, the myelin sheets showed severe disorganization and demyelination, with most structures appearing as densely spheroid-like structures (Figure 3A), an indication that the degenerated myelin sheaths had undergone collapse. Interestingly, administration of NGF efficiently reduced the abnormal myelin sheaths (likely attributed to clearance or phagocytosis by SCs), which were replaced by a few small newborn myelin sheaths (Figure 3A). Moreover, quantitative analysis from TEM also revealed that the PNI+NGF group had more newborn myelin, but less abnormal myelin, when compared with the PNI control group (Figure 3B-C).

We then measured the accumulation of the myelin lipid degradation products in each group through oil red O (ORO) staining 76. The myelin lipid degradation products, including myelin debris, are rich in lipids and lipid droplet, which were specifically dyed by ORO staining. Thereby, this method is an optimal staining of the myelin lipid degradation products. Our results revealed that 5 days after injury the sham group showed no signal for ORO staining while the PNI group accumulated myelin degradation products. NGF treatment significantly reduced the cumulative lipid tracers (Figure 3D and F). Similar results were also obtained for the quantity of MBP and MPZ (both are markers of peripheral type myelin), as determined by Western blotting (Figure S5).

Next, we harvested nerve segments from the lesion site in the PNI+NGF group at 1, 3 and 5 days. Nerve segments from the sham group were used as control (0 day). Analysis of EM images of the PNI+NGF samples revealed that the degenerative myelin sheathes underwent progressive disintegration and breakdown (Figure S6A-C). We also cultured sciatic nerve explants for 0, 1, 3 and 5 days in vitro (DIV). Immunohistochemistry of high magnification images showed that the cytoskeleton morphology of the sciatic nerve explants progressively generated ovoid-like or clustered structures over time (Figure S6D-F). Collectively, these results indicate that NGF treatment did not delay demyelination and axonal degeneration, but rather NGF likely accelerated the degeneration and clearance of myelin and axon.

To further explore whether NGF-mediated clearance of myelin debris selectively targeted SCs digestion, we first examined the quantity of SCs and myelin debris in each group via co-staining for GFAP (marker for immature SCs, red) and MPZ (the major structural protein marker for myelin, green). As shown in Figure 3E, MPZ was evenly distributed and co-localized with GFAP in the sham group with accumulation of large number of small puncta. Patches of myelin were also aggregated inside of the dedifferentiated SCs in the PNI group, indicating a significant delay in myelin degradation after nerve injury (Figure 3E). In the NGF-treated group, the disappearance of myelin debris became evident, which was further confirmed by comparing the MPZ positive area in the PNI group and in the PNI+NGF group (Figure 3G). In addition, myelin lipids within the cytoplasmic pocket of Schwann cells were clearly visualized in EM images in both the PNI and PNI+NGF groups (Figure 3H), suggesting that myelin debris digestion occurred in SCs during WD.

To further investigate if SCs degraded myelin debris, we added myelin debris (obtained from shredded nerve tissue) to RSC 96 cells, a cell line derived from SCs 77, 78. RSC 96 cells were then treated with or without NGF. Cells were stained for MBP, a marker of myelin debris and for S-100, a marker of SCs. The fluorescence intensity for MBP in untreated RSC cells was weak (Figure 3I). Following incubation with myelin debris in the culture medium for 24 h, MBP^+^ puncta were accumulated then attenuated in RSCs (Figure 3I, 0-48 h). Of note, the speed of attenuation of MBP^+^ fluorescence in the NGF-treated group was faster than that in the group control, leading to less myelin debris existing in the SCs after 48 h (Figure 3I-J). Taken together, these results point to that NGF treatment promoted myelin clearance by SCs.

### NGF activates autophagy to facilitate myelin degradation after nerve crush injury

**Figure 4:**
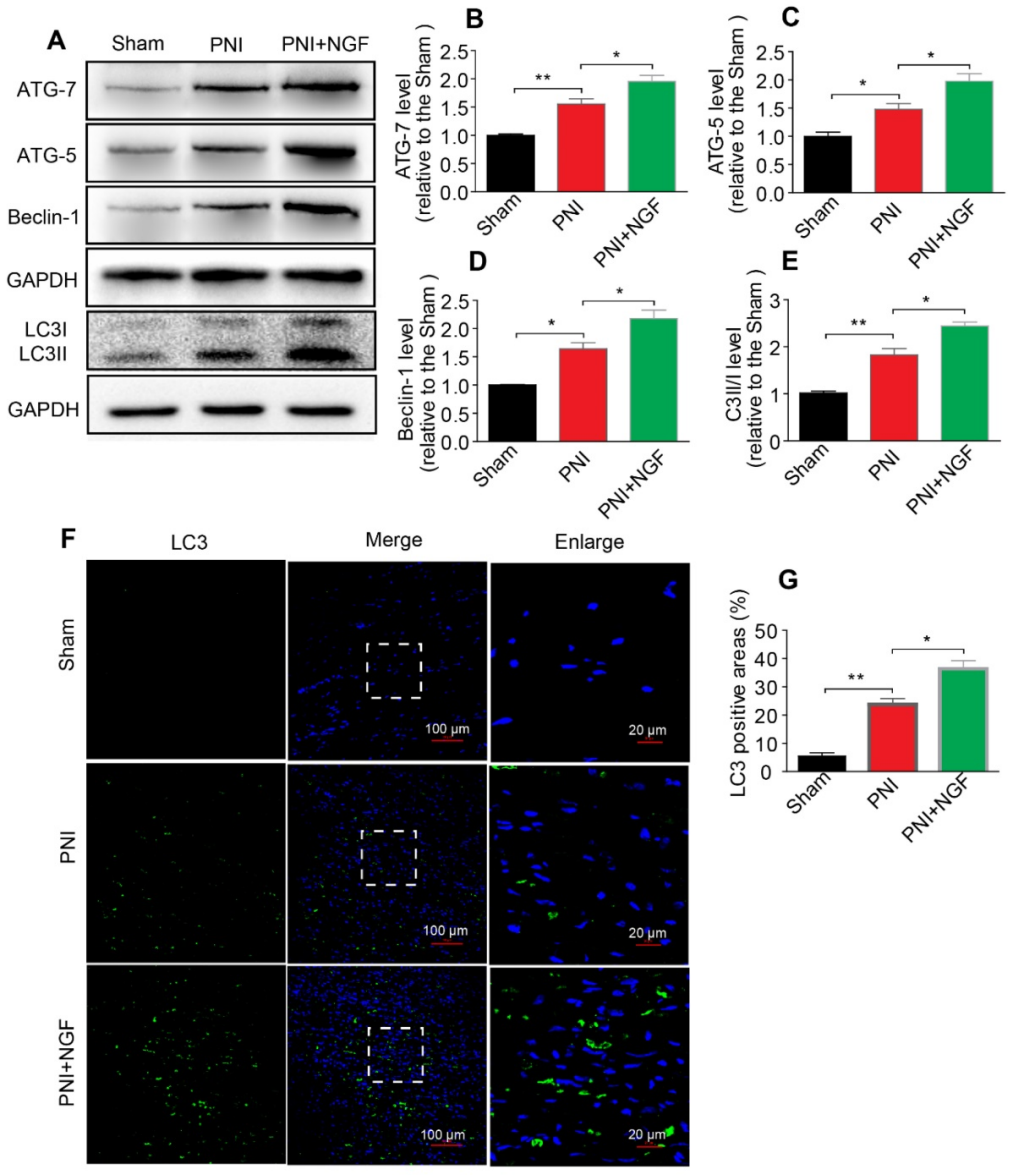
** NGF increases the level of autophagy in nerve lesions at day 5 after injury. (A)** Western blotting analysis of ATG-7, ATG-5, Beclin-1 and LC3 in sham, PNI and PNI+NGF groups at 5 days post crush.** (B-E)** Quantification of autophagy-related proteins expressed in (A). GAPDH was set as a loading control. Data are presented as the mean ± SEM; n = 3 or 4 independent experiments. ATG-7 F_(2, 6)_ = 32.47, ^**^P_sham vs PNI_ = 0.006, ^*^P_PNI vs PNI+NGF_ = 0.036; ATG-5 F_(2, 6)_ = 20.90,^ *^P_sham vs PNI_ = 0.032, ^*^P_PNI vs PNI+NGF_ = 0.041; Beclin-1 F_(2, 6)_ = 29.82,^ *^P_sham vs PNI_ = 0.027, ^*^P_PNI vs PNI+NGF_ = 0.039; LC3II/I F_(2, 9)_ =55.81,^ **^P_sham vs PNI_ = 0.0084, ^*^P = 0.031. **(F, G)** Representative images of LC3 (green) immunostaining and quantitative analysis of the average LC3 positive area in each group. n = 3 rats per group, and the results are shown as the mean ± SEM. F_(2, 6)_ = 61.36,^ **^P_sham vs PNI_ = 0.0072, ^*^P_PNI vs PNI+NGF_ = 0.041.

Autophagy participates in the clearance of peripheral myelin debris during WD 23, 26. To test whether NGF-enhanced myelin debris clearance was closely related to NGF regulation of autophagy activation, protein expressions of ATG-7, ATG-5, Beclin-1 and LC3 in each group were determined using Western blotting. As shown in Figure 4A, these autophagic proteins were increased 5d following injury and their levels were further enhanced after administrating NGF for 5 consecutive days. Statistical results of those autophagy-related proteins further confirmed our analysis (Figure 4B-E). Consistent with these findings, NGF treatment resulted in an increase in the fluorescence intensity of the autophagic marker protein LC3 in in the PNI+NGF group in comparison with the PNI group (Figure 4F-G). Collectively, these results indicated that NGF-mediated myelin clearance was accompanied by an increased activity of autophagy.

### NGF enhances autophagy through maintaining autophagic flux following contusion of the sciatic nerve

**Figure 5:**
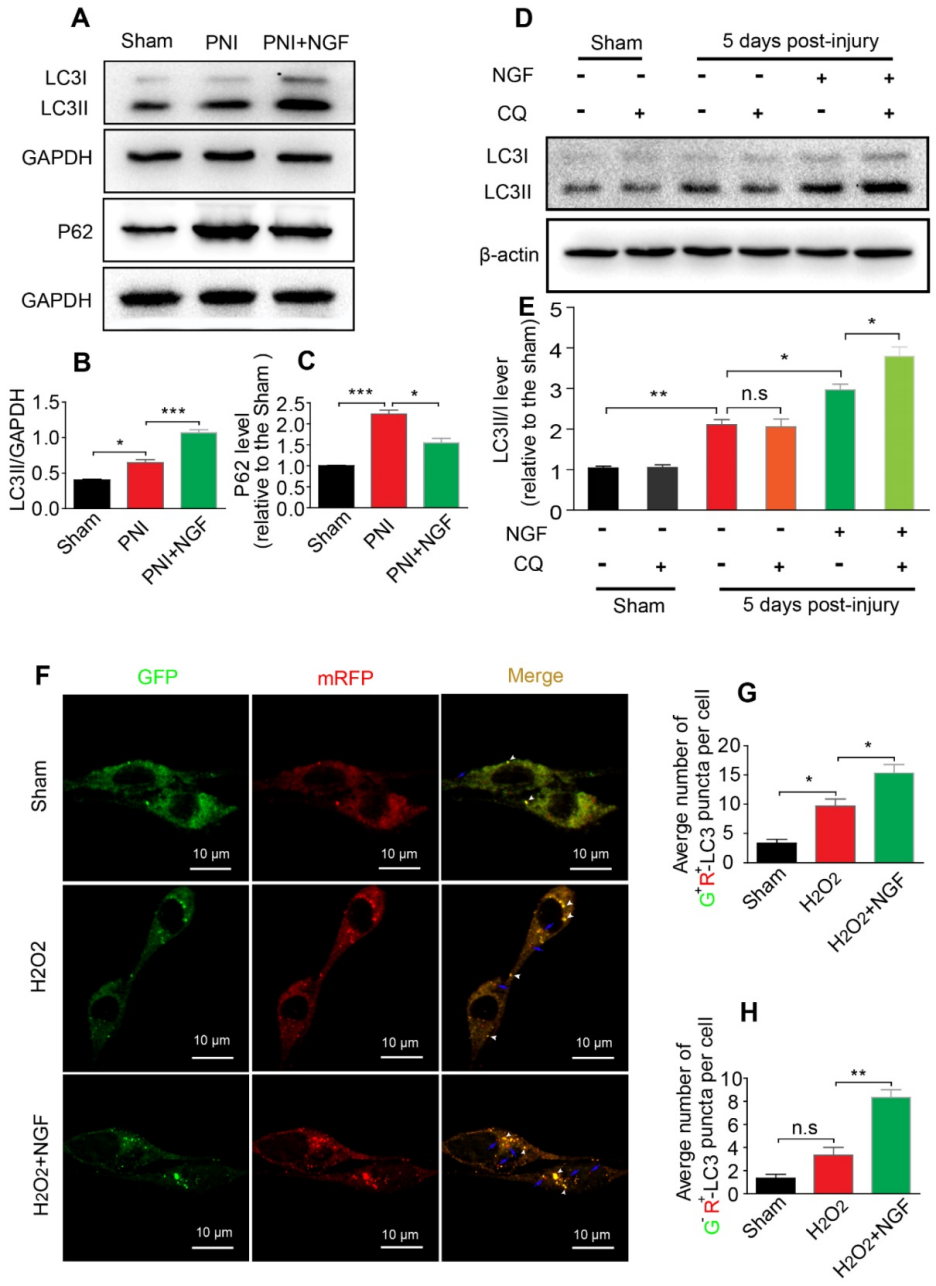
** NGF drives autophagic flux following injury in vivo and in vitro. (A-C)** Representative Western blotting and densitometric analysis of LC3II and P-62/SQSTM1 in the sham, PNI and PNI+NGF groups at 5 days post injury. Data are presented as the mean ± SEM; n = 3 independent experiments. LC3II F_(2, 6)_ = 81.92,^ *^P_sham vs PNI_ = 0.036, ^***^P_PNI vs PNI+NGF_ < 0.001; P62 F_(2, 6)_ = 53.43,^ ***^P_sham vs PNI_ < 0.001, ^*^P_PNI vs PNI+NGF_ = 0.018.** (D, E)** LC3 expression and optical density analysis in normal or injured nerves treated with NGF or CQ. β-actin was used as the loading control and for normalization. Data are the mean values ± SEM, n= 3 independent experiments. F_(5, 12)_ = 75.19,^ *^P_ PNI+NGF vs PNI+NGF+CQ_ = 0.036, *P*_PNI vs PNI+CQ_ = 0.96 (n.s), ^**^P_sham vs PNI_ = 0.0051, ^*^P_PNI vs PNI+NGF_ = 0.042. **(F)** After stably transfected with tandem-labeled mRFP-GFP-LC3 for 24 h, RSC 96 cell lines were incubated with H_2_O_2_ (100 μM) with or without NGF for another 4 h. Representative images of mRFP-GFP-LC3 vector were shown by fluorescent detection.** (G, H)** Quantitative analysis of the number of yellow (G^+^R^+^) autophagosomes and red (G^-^R^+^) autolysosomes. Data are the mean values ± SEM; Autophagosme F_(2, 6)_ = 27.03, ^*^P_sham versus PNI_ = 0.014, ^*^P_PNI versus PNI+NGF_ = 0.020; Autophagolysome F_(2, 6)_ = 39.00, *P*_sham versus PNI_ = 0.085 (n. s), ^**^P_PNI versus PNI+NGF_ = 0.0023.

The level of autophagic flux determines the autophagic fluency during the process of degrading misfolded proteins and damaged organelles 13. P62/sequestosome-1 mediates the degradation of cytosolic contents through directly binding to LC3. The changes in P62 and LC3 levels reflect the degree of autophagic flux 13, 79. P62 reduction and LC3 increase represented enhancement of the autophagic flux, namely unhindered fusion of the autophagosome and lysosome. If both proteins exhibited accumulation, this would indicate impaired degradation of the autolysosome 80. To demonstrate whether NGF increased the expression of autophagy associated-proteins ascribed to the increased formation of autolysosomes rather than decreasing their degradation, we first measured the levels of the autophagic flux marker protein P62 and its molecular chaperone LC3II by Western blotting. The result showed that the levels of LC3II and P62 became significantly increased after PNI. Interestingly, NGF treatment resulted in an increase in the expression of LC3II with a concomitant decrease in P62 (Figure 5A-C). These results demonstrated that NGF enhances autophagic flux in PNI.

To further confirm our observation, we intraperitoneally injected the lysosomal inhibitor CQ into the sciatic nerve with or without NGF treatment at day 5. 2 hours post injection, the cleavage of LC3 in the normal and injured sciatic nerves was evaluated by Western blotting. Under the condition of lysosomal inhibition with CQ, NGF treatment led to an increase in the transition of LC3I to LC3II in comparison to vehicle treatment (Figure 5D). In contrast, no significant difference exited between the PNI group and the sham group with/without CQ administration (Figure 5D-E). Taken together, these results suggest that acute PNI disrupted autophagy flux and NGF treatment promoted autophagic flux through enhancing autophagosome fusion with lysosome.

To mimic pathological changes of PNI in vitro, we treated RSC 96 cells with 100 μM H_2_O_2_ in the medium 81, 82. We used mRFP-GFP-LC3 to monitor autophagic flux. Since the GFP fluorescent intensity is more sensitive to low pH environment in autolysosomes and RFP is relatively more resistant to low pH, a decrease in the ratio of GFP^+^/mRFP^+^ signal intensity frequently indicates autophagic activity 83, 84. Control group transfected with mRFP-GFP-LC3 showed some basal level of autophagy (Figure 5F). As expected, addition of H_2_O_2_ increased the number of RFP^+^/GFP^+^ dots (white arrowheads) and these effects were further enhanced in cells treated with 50 ng/ml NGF (Figure 5F-G). NGF treatment also significantly increased the number of RFP^+^/GFP^-^ dots (blue arrows) compared with only H_2_O_2_ stimulated group (Figure 5F and H). These results demonstrated that NGF treatment increased the conversion from autophagosomes to autolysosomes to rescue the impaired autophagy flux after cell injury.

### NGF-induced autophagy activation in dedifferentiated SCs contributes significantly to myelin clearance and nerve regeneration

**Figure 6:**
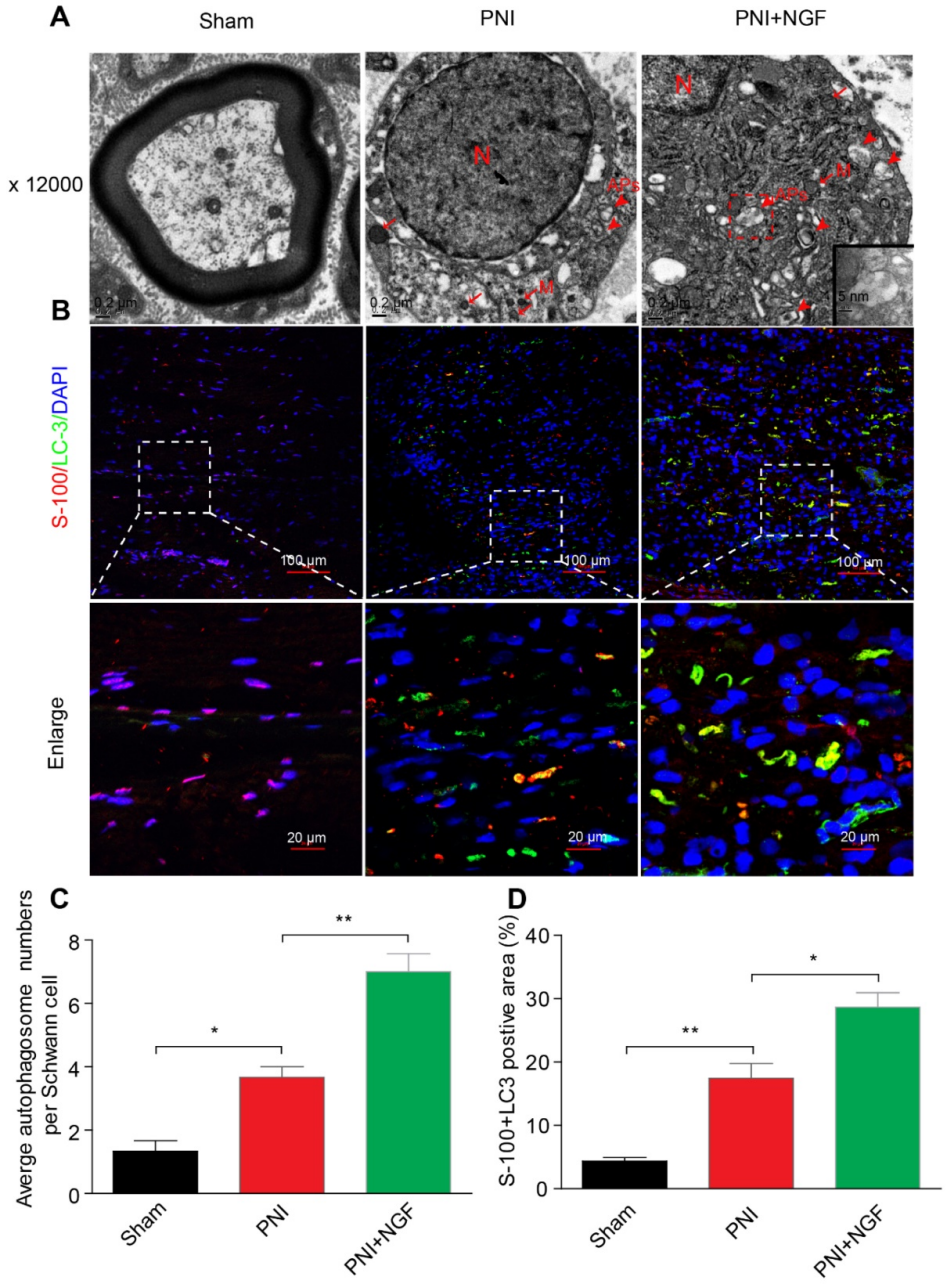
** NGF mediates the enhancement of autophagy in SCs. (A)** Transmission electronic microscopy images show numerous autophagosomes in the sham, PNI and PNI+NGF groups on postoperative day 5. APs: autophagosome (arrowheads); M: mitochondria (arrow); N: nucleus. The autophagosome is shown at high magnification in the inset. **(B)** Double immunofluorescence staining of LC3 protein (green) with S-100 positive dots (red) was detected in all groups at P5. Nuclei are counterstained with DAPI (blue). **(C)** Qualitative analysis of the average number of autophagosomes per Schwann cell from (A). Data are presented as the mean ± SEM; n = 5 rats per group. F_(2, 12)_ = 43.80, ^*^P_sham vs PNI_ = 0.015, ^**^P_PNI vs PNI+NGF_ = 0.0082.** (D)** Quantification of the percent of S-100 colocalization with LC3 in the lesion area of sciatic nerves from (B). Data are presented as the mean ± SEM; n = 3 rats per group. F_(2, 6)_ = 40.08,^ **^P_sham vs PNI_ = 0.0074, ^*^P_PNI vs PNI+NGF_ = 0.038.

SCs are responsible for myelin encapsulation to promote axonal survival and growth 85. To determine whether NGF-medicated autophagy activation and an increase in the autophagic flux following PNI was originated from SCs in vivo, we first analyzed the morphological signatures of autophagy in SCs by TEM. Autophagosomes (APs) are distinctly visible under TEM as 2 parallel membrane layers (bilayers) wrapping the substrate, which easily distinguish with the myelin membrane 86. We used this criterion to quantitate the number of APs in our experiments. As shown in the EM micrographs of transverse ultra-thin sections, APs were observed within the SCs in both the PNI group and the PNI+NGF group (arrowheads, Figure 6A). Interestingly, the average number of APs in the PNI+NGF group was nearly two-folds of that in the PNI group (Figure 6C).

In a parallel set of experiments, double immunofluorescence staining for S-100 (a marker for SCs) and LC-3 (a marker for autophagy) showed that a large number of LC-3 puncta were colocalized with S-100 in the PNI+NGF group, which were seldom seen in the PNI group. This phenomenon became more pronounced at higher magnification (Figure 6B). Quantitative results indicated that NGF treatment nearly doubled the positive area of LC3+S-100 compared to PNI (Figure 6D). These results indicate that NGF enhanced activation of autophagy and autophagic flux in SCs during WD.

### Macrophages also plays an important role in remyelination and nerve regeneration

To investigate if macrophages participated in myelin removal during peripheral nerve regeneration, we used SiO_2_ 87 or Cyclosprin A 88 to suppress macrophage activity. Following PNI, we injected SiO_2_ or Cyclosprin A at the injury sites, with or without co-administration of NGF, for 5 days. The animals were then sacrificed and sciatic nerve samples were extracted to examine the presence of macrophages and myelin clearance at the injury site (Figure S7). The presence of macrophages was detected by staining with a specific antibody against CD68, a marker for macrophages, and myelin debris was visualized with oil red O (ORO) and MPZ staining as in Figure 3D-E. Our results showed that SiO_2_ or Cyclosprin A, with or without administration of NGF, reduced the presence of macrophages at the injury site (Figure S7A and D). However, the macrophage inhibitors did not affect the amount of myelin debris as detected by either ORO (Figure S7B and E) or MPZ (Figure S7C and F) or MPZ-positive area without NGF co-injection. Yet, interestingly, even in the presence of these inhibitors, NGF treatment resulted in significant decrease in myelin debris (Figure S7B-F). These data suggest that macrophages unlikely contribute to the early phase of myelin destruction and clearance following PNI.

To investigate if macrophages played a role in remyelination and nerve rehabilitation, we prepared PNI animals that were injected SiO_2_ or Cyclosprin A at the injury sites, with or without co-administration of NGF. 14 days following PNI, we measured the number of macrophages (CD68), nerve rehabilitation (NF-200), myelin status (both MBP signals and #s of myelin sheathes) (Figure S8). At this stage, as compared with control, we did not see a difference in the numbers of macrophages in animals treated with SiO_2_ or Cyclosprin A, with or without co-administration of NGF (Figure S8A and D). However, macrophage inhibitors resulted in a significant reduction in nerve rehabilitation as measured by NF-200 signal intensity without NGF co-injection (Figure S8B and E). Interestingly, NGF treatment increased the NF-200 signals in the two samples treated with macrophage inhibitors to a level similar to the control sample that was treated with NGF (Figure S8B and E). The effect of SiO_2_ or Cyclosprin A on myelin status (MBP signals and #s of myelin sheathes) showed a trend parallel to NF-200 signal. i.e. without NGF treatment the macrophage inhibitors alone reduced both MBP signals (Figure S8B and F) and #s of myelin sheathes (Figure S8C and G). Again, the deficits induced by the inhibitors dissipated when co-treated with NGF (Figure S8B, F and C, G).

Taken together, our data point to that macrophages may not contribute significant to the early phase of myelin destruction and clearance following PNI. However, they do play an important role in remyelination and axonal regeneration in the absence of exogenous NGF treatment.

### The p75^NTR^/AMPK/mTOR signaling pathway is responsible for NGF-induced autophagy enhancement in SCs

**Figure 7:**
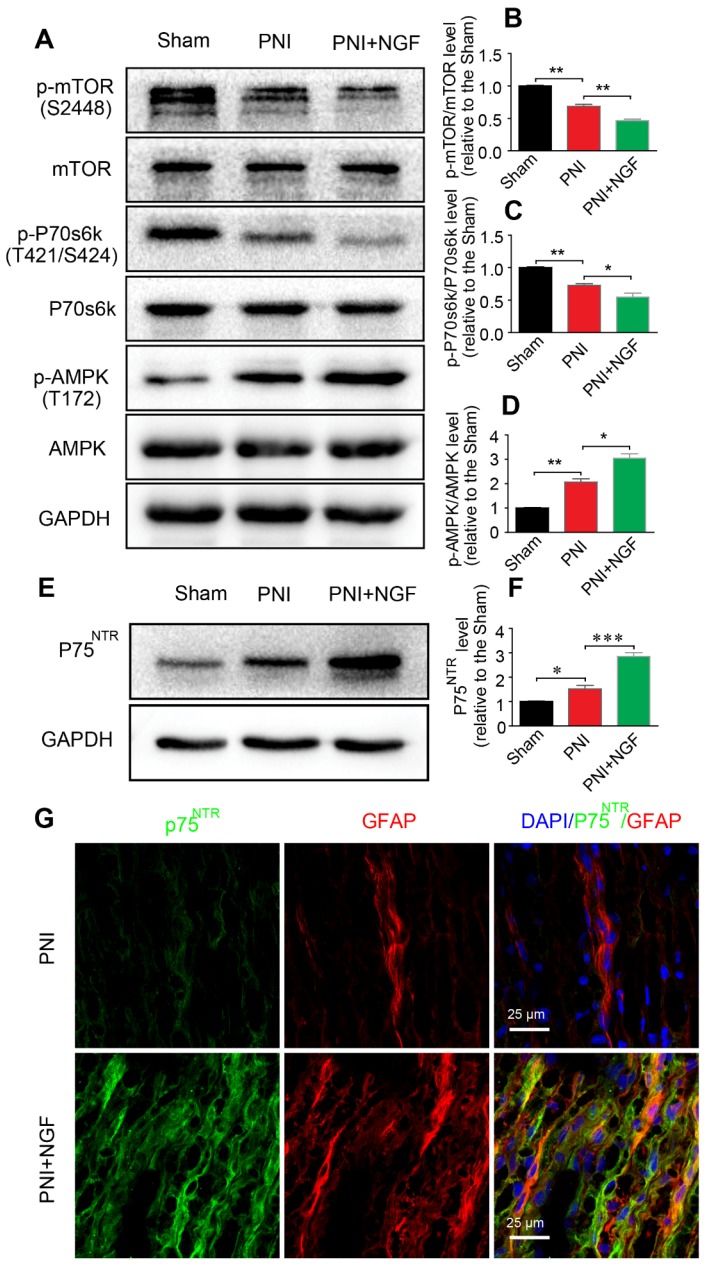
** NGF activates autophagy in SCs via the p75^NTR^/AMPK/mTOR pathway. (A)** Protein expression of *p*-AMPK (T172), AMPK, *p*-mTOR (S2448), mTOR, *p*-p70s6k (T421/S424), p70s6k in the sham, PNI model and PNI+NGF groups at 5 days post-injury; **(B-D)** Quantitative analysis of the *p*-AMPK/AMPK, *p*-p70s6k/p70s6k and* p*-mTOR/mTOR protein in each group. Data are presented as the mean ± SEM; n = 3 independent experiments.* p*-mTOR/mTOR F_(2, 6)_ = 134.50, ^**^P_sham vs PNI_ = 0.0051, ^**^P_PNI vs PNI+NGF_ = 0.0079; *p*-p70s6k/p70s6k F_(2, 6)_ = 34.06, ^**^P_sham vs PNI_ = 0.0054, ^*^P_PNI vs PNI+NGF_ = 0.042;* p*-AMPK/AMPK F_(2, 6)_ = 57.27, ^**^P_sham vs PNI_ = 0.0073, ^*^P_PNI vs PNI+NGF_ = 0.025. **(E, F)** Immunoblots and quantification of p75^NTR^. Data are presented as the mean ± SEM; n = 5 independent experiments. P75^NTR^ F_(2, 12)_ = 62.17, ^*^P_sham vs PNI_ = 0.035, ^***^P_PNI vs PNI+NGF_ < 0.001. **(G)** Immunostaining of frozen sciatic nerve sections of the PNI and PNI+NGF groups with anti-GFAP (red) and anti-p75^NTR^ (green) antibodies. Nuclei were counter-stained with DAPI (blue).

p75^NTR^ is highly expressed in SCs 89 and NGF activates p75^NTR^ to stimulate downstream signaling pathways^90^. AMPK/mTOR signaling has also been demonstrated to enhance autophagy to confer a neuroprotective effect against focal cerebral ischemia 91. AMPK upregulates autophagy through inhibiting mTOR activation 92. To determine whether NGF-mediated autophagy in SCs during WD was modulated by the p75^NTR^/AMPK/mTOR signaling, we examined the level of *p*-AMPK, *p*-p70s6k and *p*-mTOR after 5 days in sciatic nerves of injured rats by Western blotting. In comparison with the sham group, the ratio of *p*-AMPK/AMPK increased while the ratios for both *p*-mTOR/mTOR and *p*-p70s6k/p70s6k decreased after PNI. The effect was further enhanced by NGF treatment in the PNI+NGF group (Figure 7A-D).

To evaluate whether NGF regulated AMPK signaling through binding to p75^NTR^, we measured the protein levels of p75^NTR^ in each group by Western blotting. The results showed that, compared with the sham group, the p75^NTR^ protein level was significantly increased after PNI. The level of p75^NTR^ was further increased by NGF treatment in the PNI+NGF group (Figure 7E-F). Immunostaining showed that p75^NTR^ immunoreactivity was predominantly co-localized with the GFAP- positive area (Figure 7G), indicating that there was an increase in the p75^NTR^ expression in SCs in the injured nerve section receiving NGF treatment. Together, these results support that NGF increases autophagy likely through activation of the p75^NTR^/AMPK/mTOR pathway.

### Inhibition of p75^NTR^ but not Trk A specifically attenuates SC-mediated myelin degradation and impedes axonal remyelination

**Figure 8:**
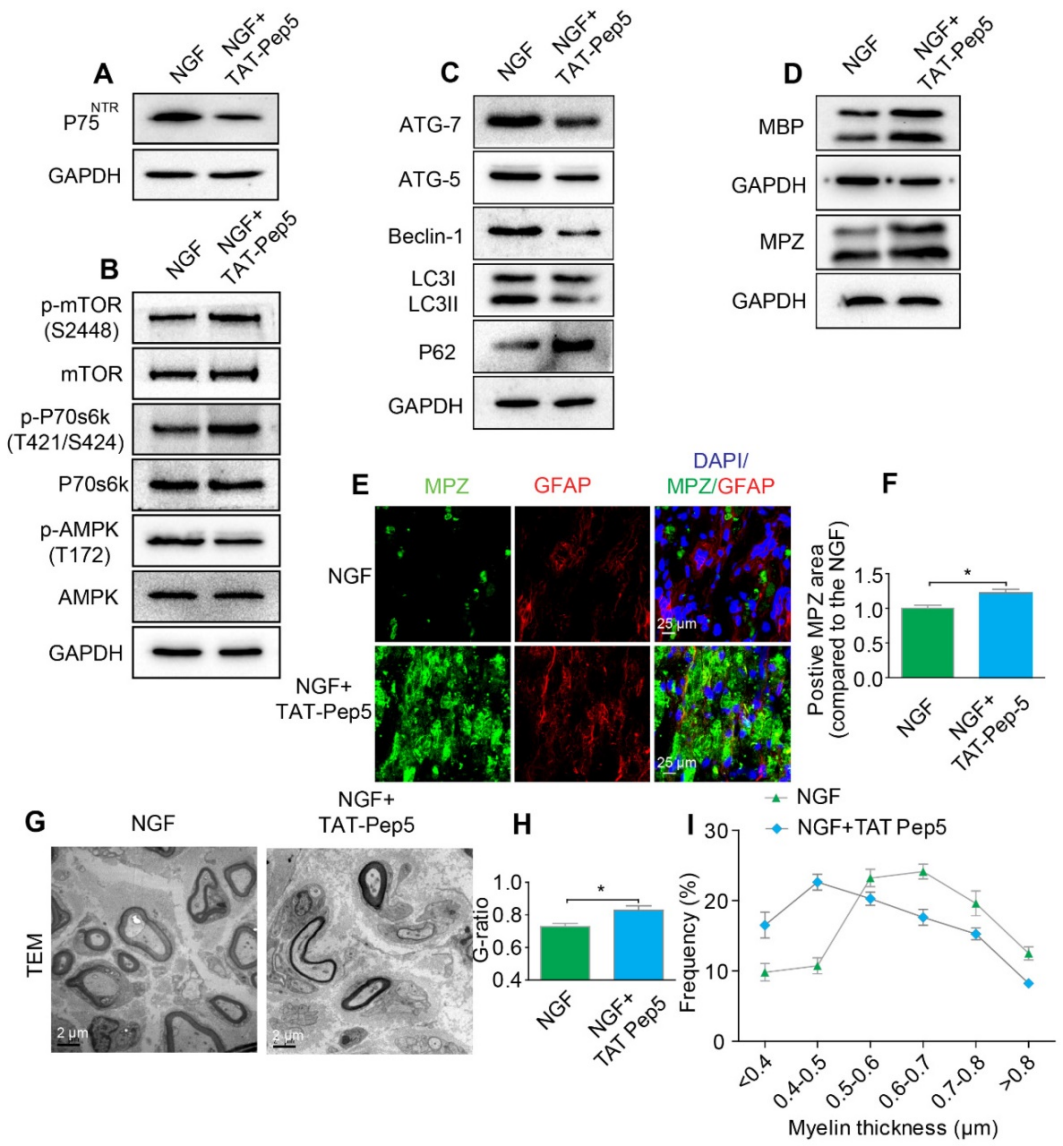
** Inhibition of p75^NTR^ reduces NGF-medicated autophagy and delays myelin clearance and axonal remyelination after sciatic nerve crush injury. (A)** Western blots of p75^NGF^ in the NGF and NGF+TAT-Pep 5 groups 5 days after injury. **(B)** Changes in the *p*-AMPK/AMPK, *p*-p70s6k/p70s6k and *p*-mTOR/mTOR ratios in each injured nerve. **(C)** Immunoblotting and quantitative analysis of ATG-7, ATG-5, Beclin-1 P62 and LC3 in the two groups. **(D)** The protein expression of MBP and MPZ in each group at 5 days post-injury. Quantitative analysis and statistical difference of western blotting results in these two groups were listed in table 2. Data are the mean values ± SEM; n = 3 or 4 independent experiments. **(E, F)** Immunofluorescence images and quantification of MPZ and GFAP signals in ipsilateral nerves 5 days after injury in NGF or NGF+TAT-Pep treatment rats. Data are the mean values ± SEM; n = 3 rats per group. MPZ ^*^P_NGF vs NGF+TAT-Pep5_ = 0.043, t = 3.205, d.f. = 4. **(G)** Representative TEM images of 14 day sections in each experimental group. Scale bar: 20 μm (HE), 2 μm (TEM) and 25 μm (Immunofluorescence). **(H-I)** Quantification of the distribution of myelin thickness, G-ratio. Data are represented as the means ± SEM; n = 3 rats per group. G-ratio ^*^P_NGF vs NGF+TAT-Pep5_ = 0.043, t = 2.938, d.f. = 4.

NGF exerts its cellular effects through two known receptors, p75^NTR^ and TrkA. If NGF activated AMPK signaling through p75^NTR^ rather than Trk A, we predicted that inhibition of p75^NTR^ by pharmacological methods would prevent SCs from effectively digesting myelin lipids and further lead to impairment of the remyelinating processes following PNI. To test our hypothesis, PNI rats were co-administered with NGF plus TAT-Pep5 93 (a p75^NTR^ inhibitor, 40 μg/0.2 mL/per animal) or NGF plus GW441756 (a TrkA inhibitor, 10 mg/kg) 94. As expected, TAT-Pep5 treatment significantly attenuated p75^NTR^ expression as compared to that of the only NGF treatment group (Figure 8A). Consistently, the effects of NGF on the downstream pathway of AMPK activation was abrogated by TAT-Pep5, as evident by the decreasing ratio of *p*-AMPK/AMPK and increasing expression of *p*-p70s6k/p70s6k and *p*-mTOR/mTOR (Figure 8B, Table 2). However, in PNI rats treated with NGF+GW441756, the ratios for *p*-AMPK/AMPK, *p*-p70s6k/p70s6k and *p*-mTOR/mTOR showed no significant difference with those of NGF treatment only (Figure S3A-D).

Furthermore, administration of TAT-Pep5 markedly promoted P62 expression and inhibited increased in the levels of ATG-7, ATG-5, and Beclin-1 proteins and the LC3-II/LC3-I ratio (Figure 8C, Table 2), while injection of GW441756 had no significant effect on the expression of these proteins (Figure S3E-I). Importantly, compared to the NGF only treatment group after sciatic nerve contusion, TAT-Pep5 significantly impeded the ability of SCs to perform myelin debris clearance (Figure 8D-F, Table 2) and axonal regeneration and remyelination (Figure S2, Figure 8G-I). But this effect was not seen in the NGF + GW441756 treated rats (Figure S3J-P). Together, these data suggest that NGF signaled through p75^NTR^, but not TrkA, to activate autophagy in SCs and facilitate myelin debris clearance and remyelination after PNI.

### Inhibition of the AMPK activation partially abolishes NGF-mediated autophagic myelin degradation in SCs during nerve regeneration

**Figure 9:**
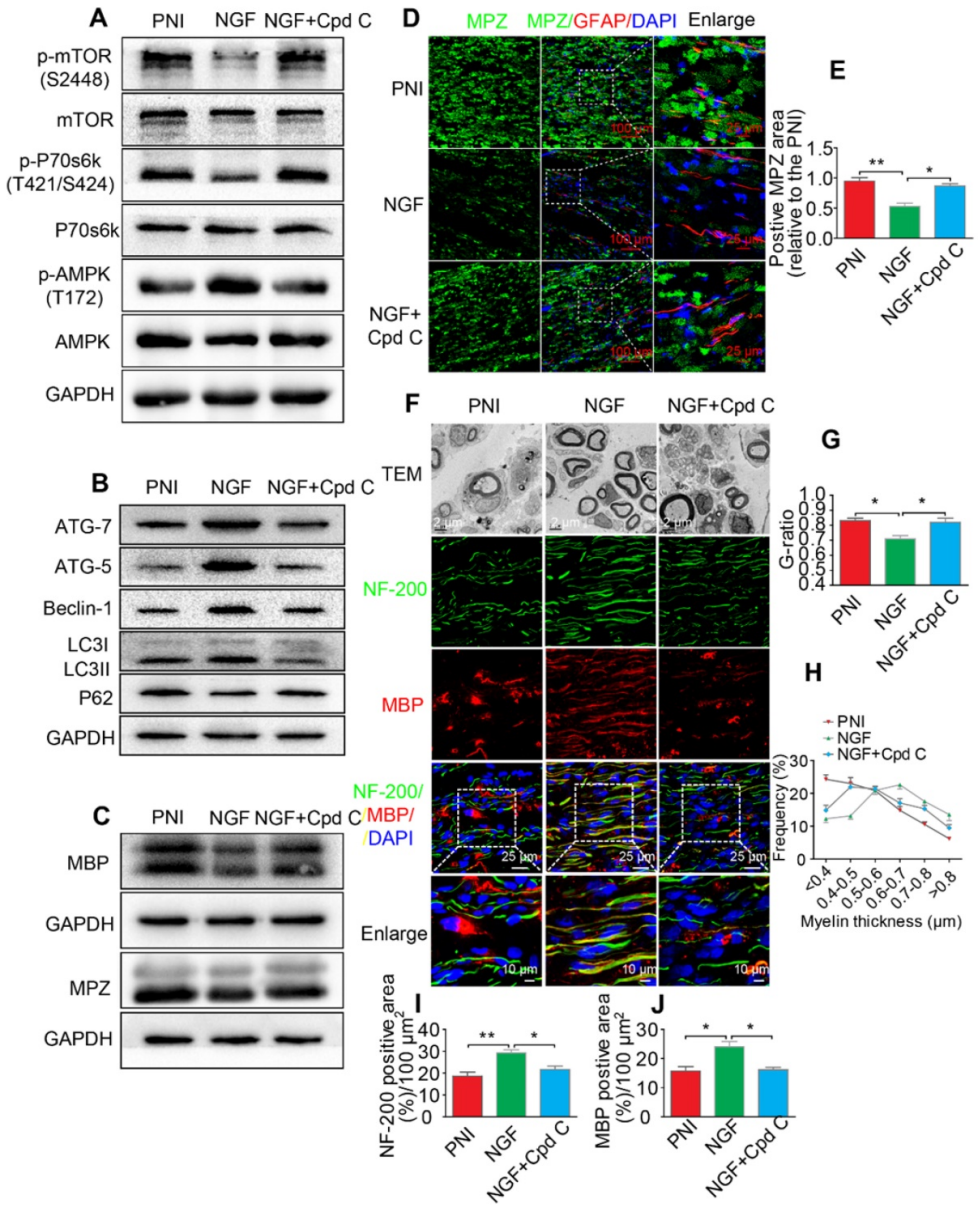
** Inhibition of AMPK significantly attenuates NGF-induced autophagic activities, myelin clearance and neural regeneration. (A)** The ratios of *p*-AMPK/AMPK, *p*-p70s6k/p70s6k and *p*-mTOR/mTOR were evaluated by western blotting in PNI, NGF and NGF+Cpd C rat sciatic nerve tissue lysates at 5 days post-injury. **(B)** Autophagy related proteins expression (including ATG-7, ATG-5, Beclin-1 P62 and LC3) were detected through western blotting. **(C)** Representative immunoblots for MBP and MPZ in each group of rats. Quantitative data and statistical analysis of western blotting results in these three groups were showed in table 3. Data are presented as the mean ± SEM; n = 3 or 4 independent experiments. **(D, E)** Co-immunofluorescence images and quantification showing MPZ (green) and GFAP (red) in injured sciatic nerve at day 5. Nuclei are blue (DAPI). Original scale bar = 100 µm and close-up scale bar = 25 µm. Data are presented as the mean ± SEM; n = 3 rats per group. MPZ F(2, 6) = 18.89, ^**^P_PNI vs NGF_ = 0.0053, ^*^P_NGF vs NGF+Cpd C_ = 0.015. **(F)** TEM images and double staining for MBP (red)/NF-200 (green) of sections from the injured sciatic nerve in each rat group at 14 days post-injury. Nuclei are blue (DAPI). **(G-J)** Analysis of G-ratio, myelin thickness distribution, and NF-200- and MBP- positive staining in each group. Data are presented as the mean ± SEM; n = 3 rats per group. G-ratio F_(2, 6)_ = 8.64, ^*^P_PNI vs NGF_ = 0.043, ^*^P_NGF vs NGF+Cpd C_ = 0.045; NF-200 F(2, 6) = 11.89, ^**^P_PNI vs NGF_ = 0.0078, ^*^P_NGF vs NGF+Cpd C_ = 0.034; MBP F(2, 6) = 10.08, ^*^P_PNI vs NGF_ = 0.041, ^*^P_NGF vs NGF+Cpd C_ = 0.043.

To define a role of AMPK signaling in NGF-mediated autophagy and its legacy effect, NGF and Cpd C - a specific AMPK inhibitor 95, were co-administered to PNI rats. Changes in the levels of *p*-mTOR, mTOR, *p*-AMPK, AMPK, *p*-p70s6k, and p70s6k were detected by Western blotting. The results showed that NGF-induced AMPK activation was significantly suppressed by Cpd C treatment (Figure 9A, Table 3). In addition, inactivating the AMPK pathway with Cpd C further blocked the elevated level of autophagy in nerve contused rats receiving NGF treatment, as evident by a decrease of the ATG-7, ATG-5, and Beclin-1 levels and the LC3-II/I ratio with a concomitant elevation of P62 (Figure 9B, Table 3).

Next, we focused on the efficiency of Cpd C in NGF-regulated myelin breakdown and clearance. Immunofluorescence and Western blotting analysis revealed that Cpd C delayed the effects of NGF in promoting myelin fragment clearance (Figure 9C-E, Table 3). We then tested whether Cpd C inhibited the effect of NGF on axonal growth and myelin regeneration. As indicated in Figure 9F, the regenerated myelin and nerve fibers were more loose, sparse and irregular in NGF+Cpd C rats compared to those of rats treated with NGF alone. Statistical analysis of the ranking of myelin thickness, the G-ratio and the signals for NF-200 and MBP areas also showed a similar effect (Figure 9G-J).

**Figure 10:**
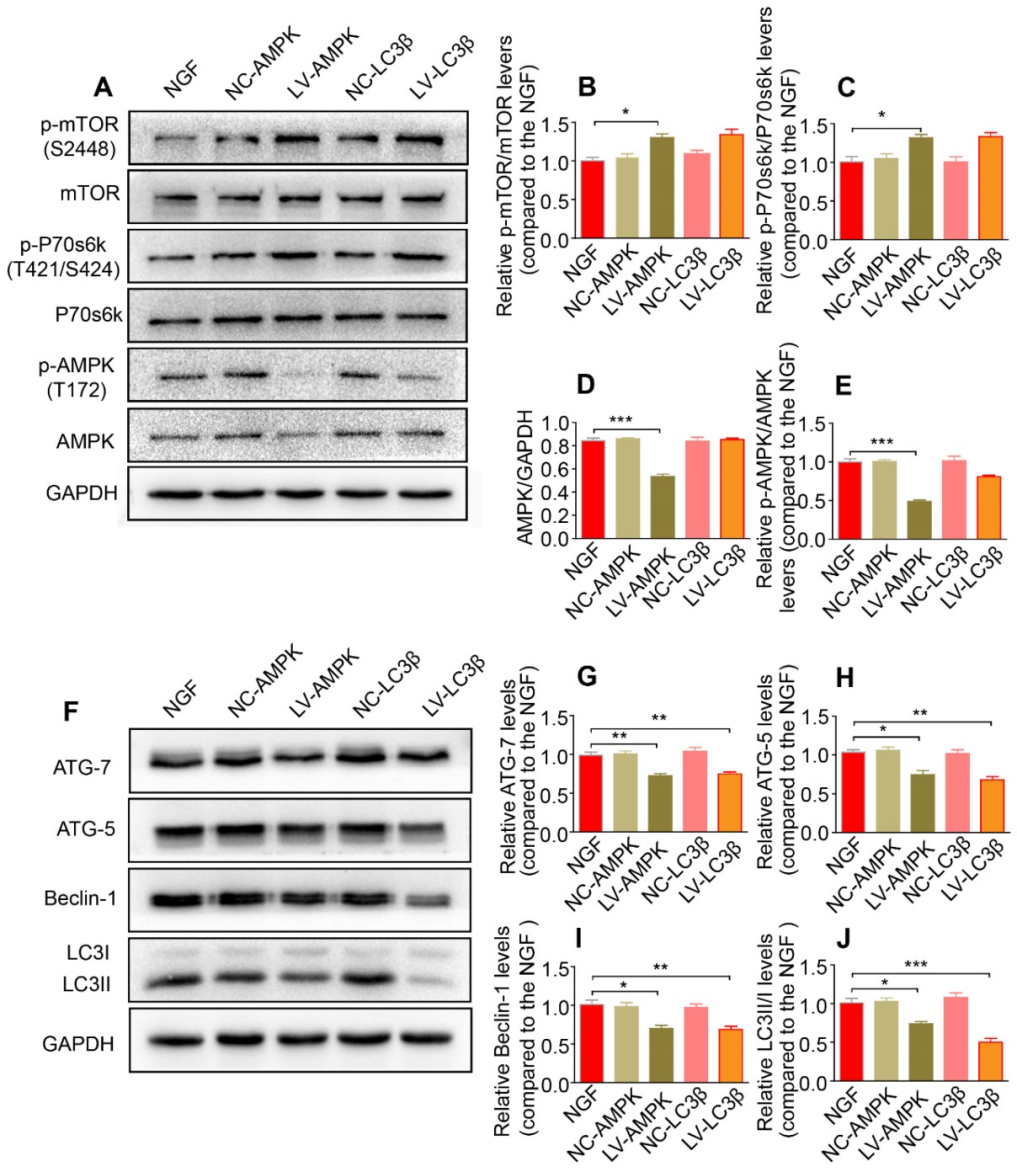
** Reducing AMPK or LC3 expression significantly inhibits the autophagy and its upstream signaling activation. (A-E)** Representative immunoblots of *p*-AMPK, AMPK, *p*-p70s6k, p70s6k, *p*-mTOR and mTOR in NGF therapeutic rats infected with/without LV-AMPK-RNAi/LV-NC_AMPK_-RNAi or LV-LC3β-RNAi/LV-NC_LC3β_-RNAi and quantification of these data. Data are the mean values ± SEM; n = 3 independent experiments. *p*-mTOR/mTOR F_(4, 10)_ = 7.99, ^*^P_NGF vs LV-AMPK_ = 0.011; *p*-p70s6k/p70s6k F_(4, 10)_ = 8.30, ^*^P_NGF vs LV-AMPK_ = 0.019; AMPK/GAPDH F_(4, 10)_ = 44.48, ^***^P_NGF vs LV-AMPK_ < 0.001; *p*-AMPK/AMPK F_(4, 10)_ = 41.67, ^***^P_NGF vs LV-AMPK_ < 0.001. **(F-J)** Autophagy related proteins (including ATG-7, ATG-5, Beclin-1 and LC3) were detected by western blotting and quantified their expression in those five groups. Data are presented as mean ± SEM; n = 3 independent experiments. ATG-7 F_(4, 10)_ = 17.48, ^**^P_NGF vs LV-AMPK_ = 0.0054, ^**^P_NGF vs LV-LC3β_ = 0.0070; ATG-5 F_(4, 10)_ = 16.48,^ *^P_NGF vs LV-AMPK_ = 0.017, ^**^P_NGF vs LV-LC3β_ = 0.0028; Beclin-1 F_(4, 10)_ = 11.56,^ *^P_NGF vs LV-AMPK_ = 0.011, ^**^P_NGF vs LV-LC3β_ = 0.0092; LC3II/I F_(4, 10)_ = 24.59,^ *^P_NGF vs LV-AMPK_ = 0.016, ^***^P_NGF vs LV-LC3β_ < 0.0001.

**Figure 11:**
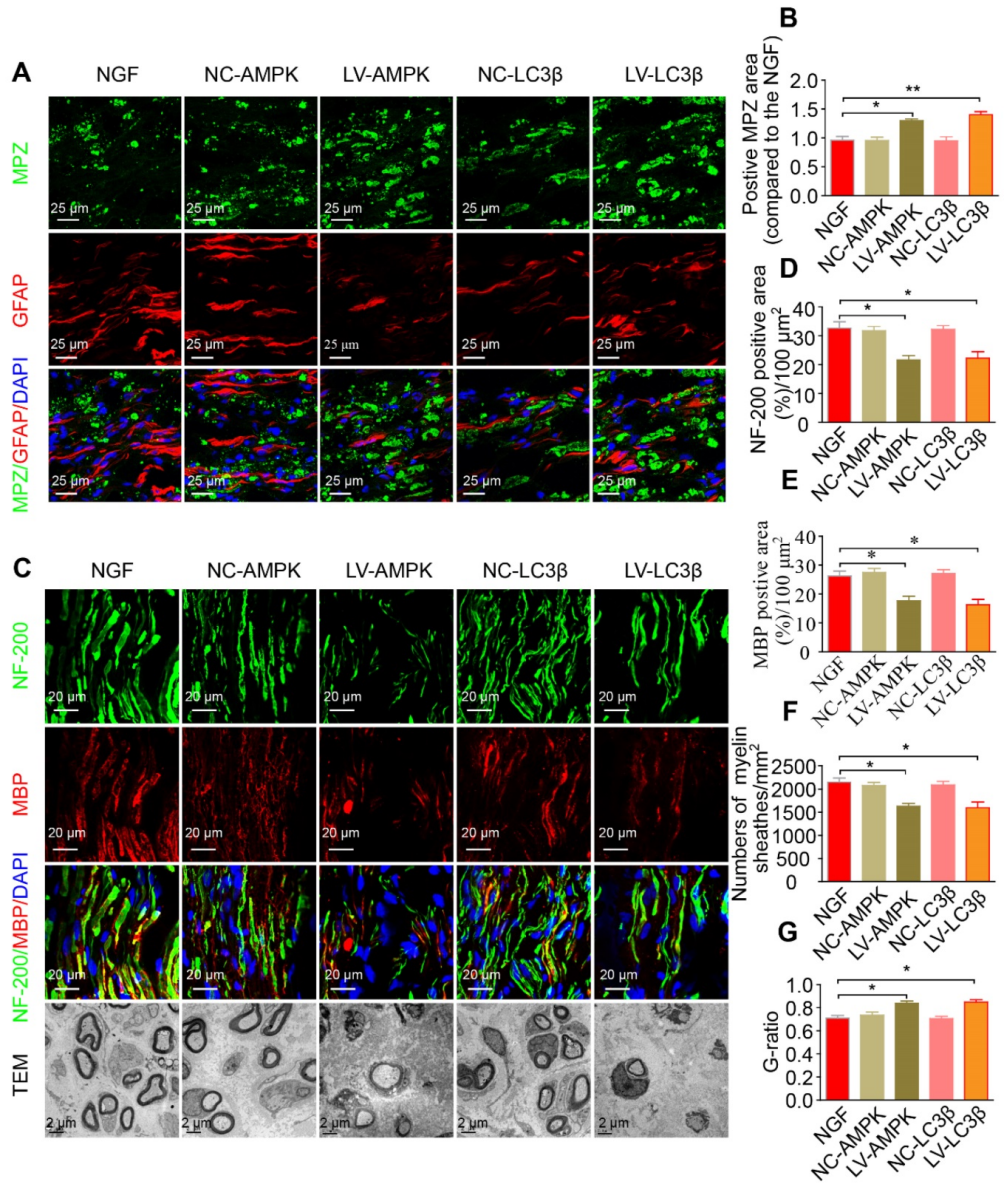
**RNAi-mediated knocking-down of AMPK impairs myelin degradation, axonal regeneration and remyelination. (A)** Co-immunostaining with anti-MPZ (green) and anti-GFAP (red) antibodies in injured sciatic nerve at day 5. Nuclei were blue (DAPI). **(B)** The positive MPZ areas in each group were calculated. Data are presented as mean ± SEM; n = 3 rats per group. MPZ F_(4, 10)_ = 12.23,^ *^P_NGF vs LV-AMPK_ = 0.020, ^**^P_NGF vs LV-LC3β_ = 0.0087. **(C)** Double-immunostaining for MBP (red)/NF-200 (green) and TEM images of sections from the injured sciatic nerve in each group rats at 14 days. Nuclei were blue (DAPI). **(D-G)** Analysis of NF-200 and MBP positive staining, numbers of myelin sheaths and G-ratio in each group. Data are presented as mean ± SEM; n = 3 rats per group. NF-200 F_(4, 10)_ = 9.77, ^*^P_NGF vs LV-AMPK_ = 0.015, ^*^P_NGF vs LV-LC3β_ = 0.030; MBP F_(4, 10)_ = 12.23, ^*^P_NGF vs LV-AMPK_ = 0.020, ^*^P_NGF vs LV-LC3β_ = 0.017; myelin numbers F_(4, 10)_ = 9.48, ^*^P_NGF vs LV-AMPK_ = 0.014, ^*^P_NGF vs LV-LC3β_ = 0.022; G-ratio F_(4, 10)_ = 10.45, ^*^P_NGF vs LV-AMPK_ = 0.013, ^*^P_NGF vs LV-LC3β_ = 0.017. Significance was determined with the unpaired t-test with Welch's correction.

Additionally, silencing AMPK gene expression through orthotopic injection (OI) of Lenti-AMPK-RNAi significantly blocked the AMPK expression and decreased the ratio of *p*-AMPK/AMPK and *p*-p70s6k/p70s6k, but also increased the expression of *p*-mTOR/mTOR (Figure 10A-E). Moreover, the downstream biological effects, including autophagic activation, myelin clearance and nerve reestablishment, were all delayed after knock-down of AMPK activation (Figure 10F-J and Figure 11). Therefore, these results provide compelling evidence that NGF activated AMPK to upregulate autophagy-mediated clearance of myelin fragments to expedite remyelination.

### Inhibition of autophagic activation significantly inhibits NGF effects on myelin phagocytosis and nerve regeneration

**Figure 12:**
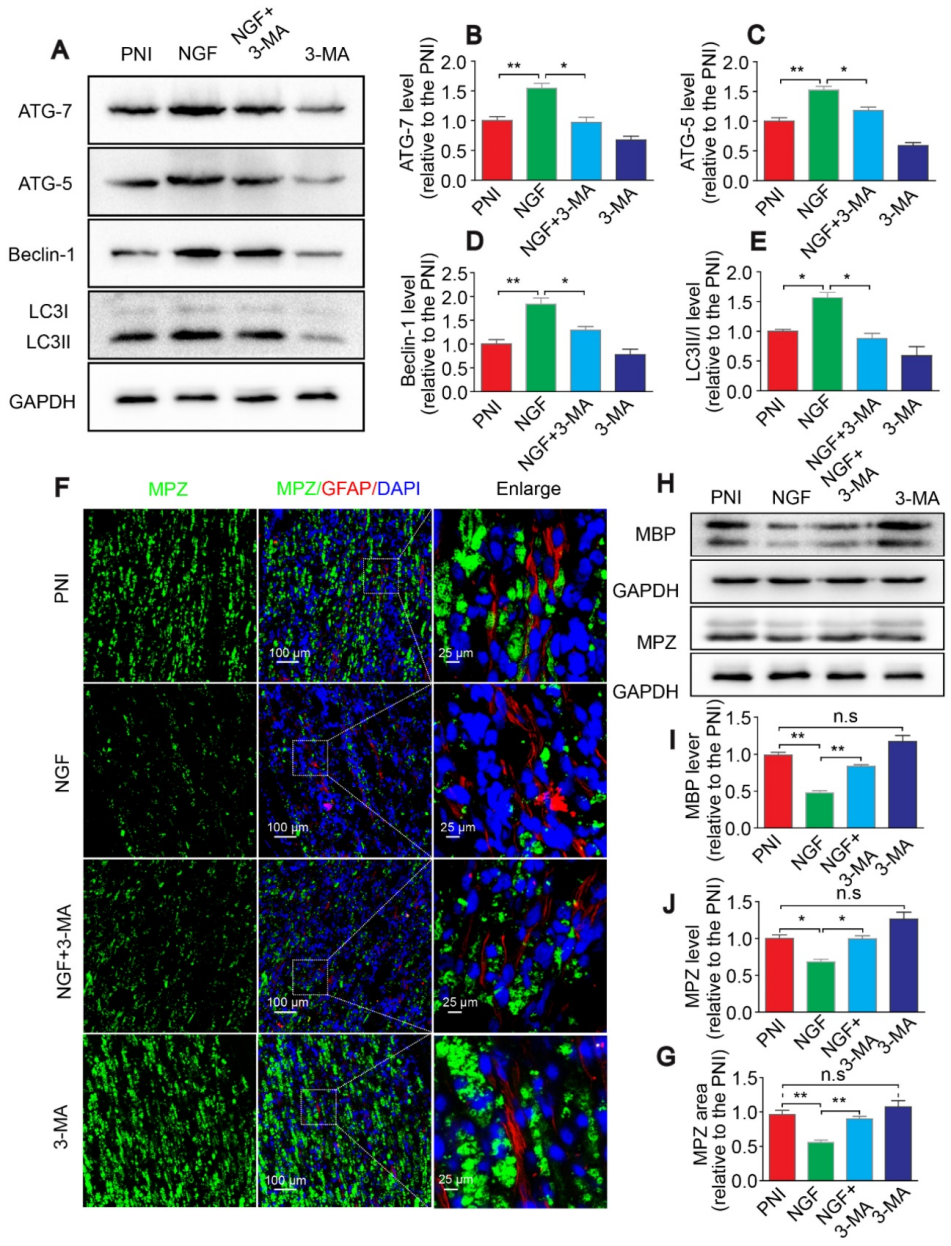
** Autophagy inhibition delays myelin degradation. (A-E)** Representative immunoblots and quantification of ATG-7, ATG-5, Beclin-1 and LC3 from sciatic nerves of the PNI, NGF, NGF+3-MA and 3-MA groups at 5 days post crush. Data are presented as the mean ± SEM; n = 3 independent experiments. ATG-7 F_(3, 8)_ = 22.16, ^**^P_PNI vs NGF_ = 0.0076, ^*^P_NGF vs NGF+3-MA_ = 0.019; ATG-5 F_(3, 8)_ = 46.66, ^**^P_PNI vs NGF_ = 0.0036, ^*^P_NGF vs NGF+3-MA_ = 0.027; Beclin-1 F_(3, 8)_ = 18.80, ^**^P_PNI vs NGF_ = 0.0075, ^*^P_NGF vs NGF+3-MA_ = 0.038; LC3II/I F_(3, 8)_ = 16.85, ^*^P_PNI vs NGF_ = 0.039, ^*^P_NGF vs NGF+3-MA_ = 0.012. **(F, G)** Representative micrographs showing double immunofluorescence with MPZ (green) and GFAP (red). Nuclei are stained with DAPI (blue) in each group. Quantitation of the MPZ positive area is also shown. Data are presented as the mean ± SEM; n = 3 rats per group. F_(3, 8)_ = 13.37, ^**^P_PNI vs NGF_ = 0.0057, ^**^P_NGF vs NGF+3-MA_ = 0.0054, *P*_PNI vs 3-MA_ = 0.39 (n.s).** (H-J)** MBP and MPZ protein levels and quantitative analysis. Data are presented as the mean ± SEM; n = 3 independent experiments. MBP F_(3, 8)_ = 44.72, ^**^P_PNI vs NGF_ = 0.0074, ^**^P_NGF vs NGF+3-MA_ = 0.0056, *P*_PNI vs 3-MA_ = 0.11 (n.s); MPZ F_(3, 8)_ = 31.98, ^*^P_PNI vs NGF_ = 0.036, ^*^P_NGF vs NGF+3-MA_ = 0.012, *P*_PNI vs 3-MA_ = 0.090 (n.s).

**Figure 13:**
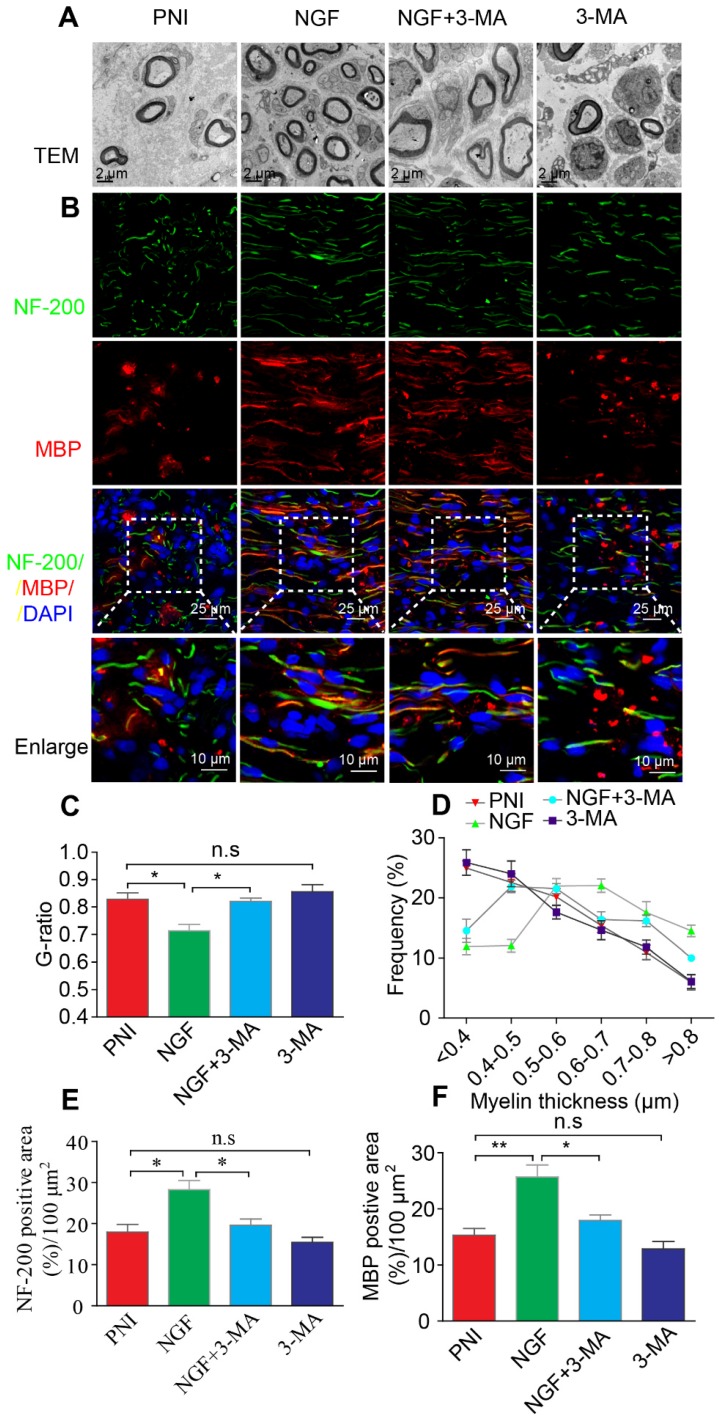
** Autophagy inhibition suppresses nerve regeneration. (A, B)** Electron micrographs and co-immunofluorescence of NF-200 (green) and MBP (red) analysis were performed in the four groups at 14 days after PNI. Nuclei are stained with DAPI (blue). **(C-F)** Statistical analysis of the G-ratio, distribution of myelin thickness, and NF-200 and MBP positive staining areas on the proximal nerve lesions in each group. Data are presented as the mean ± SEM; n = 3 rats per group. G-ratio F_(3, 8)_ = 8.23, ^*^P_PNI vs NGF_ = 0.039, ^*^P_NGF vs NGF+3-MA_ = 0.040, *P*_PNI vs 3-MA_ = 0.49 (n.s); NF-200 F_(3, 8)_ = 10.17, ^*^P_PNI vs NGF_ = 0.042, ^*^P_NGF vs NGF+3-MA_ = 0.048, *P*_PNI vs 3-MA_ = 0.35 (n.s); MBP F_(3, 8)_ = 13.91, ^**^P_PNI vs NGF_ = 0.005, ^*^P_NGF vs NGF+3-MA_ = 0.039, *P*_PNI vs 3-MA_ = 0.058 (n.s).

To further confirm that NGF acts through autophagy in SCs to enhance myelin breakdown and remyelination, we used a classical autophagy inhibitor, 3-MA to block NGF-induced autophagy 67. Western blotting analysis confirmed that the pharmacologic inhibitor reduced the levels of ATG-7, ATG-5, and Beclin-1 and the LC3-II/I ratio dramatically, compared to animals treated with NGF alone (Figure 12A-E). Immunoblotting and immunostaining analyses of longitudinal sections of contusive sciatic nerves showed that administration of 3-MA to the NGF group, but not the PNI group, significantly delayed the myelin breakdown and suppressed residual myelin- related protein clearance (including MBP and MPZ, Figure 12F-G). Next, we tested the effects of 3-MA on the therapeutic response. Using TEM and immunofluorescence double staining, we found that inhibition of autophagy with 3-MA in the NGF group led to a notable reduction in the myelin sheath thickness and the number of regenerated axons. Nevertheless, no significant difference existed between the PNI and PNI+3-MA groups (Figure 13).

Additionally, we employed a lentiviral system to deliver shRNA-LC3β in vivo. As illustrated in Figure 10 and Figure 11, after injection with Lenti-LC3β-RNAi, the NGF-treated rats exhibited significant downregulation of autophagy levels and reduction of myelin clearance and nerve regrowth. Together, we thus conclude that NGF-induced autophagic activation served as a protective mechanism in myelin clearance and nerve regeneration.

### NGF promotes myelin phagocytosis and clearance in primary Schwann cells

To confirm if NGF had a similar effect in phagocytosis myelin debris in primary SCs, we purchased highly pure (>98%) rat primary Schwann cells (SCs, Cat# EM1010) from Kerafast, Inc. (Boston, MA, USA). Per the supplier, these cells express S100, p75^NTR^ (see https://www.kerafast.com/product/3171/rat-primary-schwann-cells), both are markers for Schwann cells. We also followed published protocols 65, 66 and purified peripheral nerve system (PNS) myelin debris. We conjugated the myelin preparations with pHrodo™ Red, succinimidyl ester (pHrodo, Thermo Fisher Scientific, P36600). The advantage of using pHrodo™ Red to label myelin debris is that pHrodo™ Red will only emit red fluorescent signals in acidic pH environments such as the cellular interior 65, 66. Therefore, the pHrodo™ Red signals can be only detected by fluorescent microscopy following internalization of pHrodo™ Red-myelin debris into SCs. On the other hand, un-internalized pHrodo™ Red-myelin debris will not be seen under the microscope. This is the basis for our new experiments.

We performed the myelin phagocytosis assay according to the well-established methods by Ben Barres group 64. Briefly, primary SCs were cultured in 6-well plates at the density of 1.00×10^6^ cells/well. The next day, cells in each well were rinsed once with phosphate buffer saline (PBS). For the control experiment, SCs were incubated with 1mL complete media supplemented with 10% Fetal Bovine Serum (FBS) and 800 μg mL^-1^ of pHrodo™ Red-myelin debris. For the NGF treatment group, NGF (final concentration: 50 ng mL^-1^) 63 was also added to the complete media containing pHrodo-conjugated myelin debris.

To define the effect of 3-MA in the decrease of myelin staining after feeding the myelin in SCs, we first used NGF (50 ng mL^-1^) to stimulate the uptake of pHrodo™ Red-myelin debris by SCs for 6 hrs. We then added 5 mM (final concentration) of 3-MA 67 (Aladdin, M129496) into the media to block autophagic activities.

**Figure 14:**
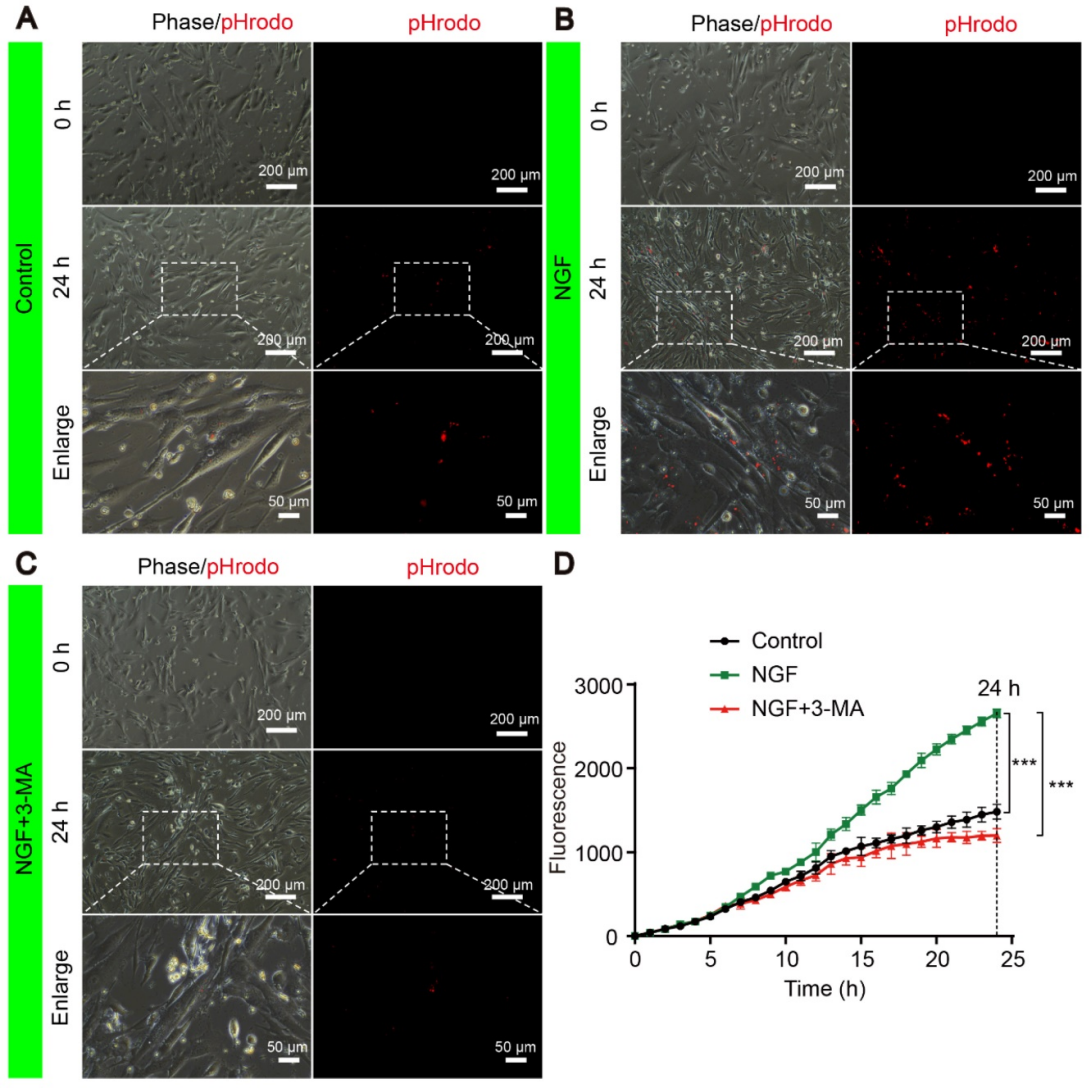
** NGF enhances myelin phagocytosis in primary Schwann cells.** (A-C) Cells were purchased and cultured as described in Materials and Methods. Cells were treated with either vehicle control (A), or 50 ng mL^-1^ NGF (B) or 50 ng mL^-1^ NGF for 6 hrs followed by the addition of 3-MA (C). Representative phase and fluorescent images of primary SCs at 0 h and 24 h for each condition are shown. An magnified inset for each treatment group is also presented to show the pHrodo-labeled myelin debris were inside of primary SCs.** (D)** The signals of integrated fluorescence intensity of internalized pHrodo-labeled myelin debris were measured and shown. n = 5 picture frames for each group per time point. Data are presented as mean ± SEM. T = 24 h F_(2, 12)_ = 118.84, ^***^P_control vs NGF_ < 0.001, ^***^P_NGF vs NGF+3-MA_ < 0.001.

The signals of internalized pHrodo™ Red-myelin debris i.e. red fluorescence in these primary SCs, were detected and captured by live cell imaging at an 1 hr interval for 24 hrs using a Nikon ECLIPSE Ti microscope (10× objective lens) (Nikon, Japan). For each sample, we took 5 images of ~from random areas of the 6 well plates and calculated the integrated fluorescence intensity using the ImageJ software. Representative live-cell images of SCs at 0 and 24 h were shown for the control (Figure 14A), NGF (Figure 14B) and NGF+3-MA groups (Figure 14C). An enlarged inset for each group was also show to demonstrated that the red fluorescent pHrodo™ Red-myelin debris were indeed inside of the primary SCs.

Quantification of integrated fluorescence intensity of internalized pHrodo™ Red-myelin debris from 0 to 24 hr in each group (Figure 14D) showed that: 1) the integrated fluorescence intensity in the control group showed a progressive increase; 2) NGF treatment significantly enhanced the uptake of pHrodo™ Red-myelin debris with their fluorescent intensity nearly doubling of that in the control group at 24 hr; 3) 3-MA treatment significantly suppressed the effect of NGF on enhancing uptake of pHrodo™ Red-myelin debris. Together with results obtained with RSC 96 cells, we conclude that NGF likely acts to increase the autophagic activities to facilitate the update of myelin debris in primary SCs, which in turn contributes to nerve regeneration.

## Discussion

Using a rat PNI model, we investigated the effect of exogenous NGF in modulating myelin debris clearance during WD and examined its cellular and molecular mechanisms. As illustrated in schematic 1, the major findings of our study are: (1) administration of exogenous NGF accelerated the collapse of degenerative nerves and promoted myelin debris clearance during WD; (2) this effect was resulted from NGF-mediated autophagic enhancement and autophagic flux flows in SCs; and (3) NGF- activated autophagy in SCs at the early period of PNI was likely regulated by the p75^NTR^/AMPK/mTOR signaling pathways. Our study has thus provided a novel mechanistic insight into the robust effect of NGF in enhancing axonal regrowth and remyelination to facilitate the recovery of injured nerves.

Peripheral nerve regeneration is a physiological repair process that triggers a series of highly regulated cellular and molecular responses that govern axon elongation, remyelination and synaptic formation 96. Myelin debris originates from damaged myelin breakdown. A bulk of myelin accumulation deteriorates the nerve regenerative microenvironment and impedes remyelination 97. Thus, myelin removal is essential for nerve regeneration.

SCs, as a classical type of glial cells in the PNS, are capable of forming consecutive and multi-layered plasma membranes enwrapping large-caliber axons to reestablish the structure and function linkage between the distal nerve stump and its target organs 98. Moreover, they also create a regenerative microenvironment suitable for nerve regeneration by secreting neurotrophic factors and removing myelin debris during WD 99. Studies have pointed to that SCs-mediated myelin clearance through the entire process of WD provides an important pathway, guiding axon growth along the basal lamina tubes 100, 101. Accordingly, improving the phagocytosis capability in SCs will be a key factor in facilitating the remyelination process.

NGF has been demonstrated to exert neuroprotective and neurotrophic effects in neural survival, development, function and peripheral nerve repair 69, 70. However, it is unclear if the effect can be attributed to either NGF action on neurons or on SCs or on macrophage. To clarify this issue, we injected TrkA inhibitors (K252a, GW441756) or p75^NTR^ inhibitor (TAT-Pep 5) in vivo. We found that inhibiting NGF/TrkA in neurons had no appreciable effect in NGF-mediated nerve recovery under our experimental settings (Figure S3 and Figure S4). In contrast, suppressing p75^NTR^ activation in SCs delays axonal regrowth and remyelination (Figure 8 and Figure S3). Taken together, our results have suggested that NGF acts on p75^NTR^ to promote nerve regeneration.

Even though our present study supports that NGF effects on SCs to expedite myelin clearance and nerve regeneration following PNI, Macrophages also play an important role in modulating their phagocytic ability in the lesion area by secreting neurotrophic factors, inflammatory cytokines and chemokines 97, 102, 103. For example, macrophages secrets NGF, and NGF production by macrophages is enhanced by injury and inflammation 104, 105. However, it is relatively less studied if macrophages express NGF receptors (p75^NTR^, TrkA). In a recent paper, Williams et al have demonstrated that human macrophages appeared to express both TrkA and p75^NTR^ and NGF treatment increased membrane ruffling, calcium spiking, phagocytosis and growth factor secretion 106. Our current study has suggested that macrophages play an important role in the early phase of myelin clearance that contributes importantly to axonal regeneration. However, it is unknown at present if and how NGF acts on macrophages to impact myelin clearance and nerve regeneration.

Based on our current study, we speculate that SCs-mediated removal of the myelin debris occurs during both the first phase (0-5 days) and second phase (5-14 days) of nerve injury, myelin clearance and regeneration. On the other hand, macrophages modulate their phagocytic ability and aggregate at the site of an injured region in the first phase of injury (0-5 days) and contribute to myelin debris clearance largely in the second phase (5-14 days post-injury). Therefore, blocking macrophages with inhibitors would not impact the early phase of myelin clearance as we have demonstrated herein. These notions are consistent with our macrophage inhibitor results that activity during the first phage would not affect myelin clearance since SCs are still able to perform these tasks. It is in the second phase that macrophages together with SCs contribute significantly to the clearance of myelin debris. This is consistent with a study showing that depletion of macrophages delayed myelin destruction and clearance following nerve injury 107. We argue that exogenously administered NGF enhances the autophagic activity to increase the digestive capacity in SCs for speedy removal of myelin debris, as such, to facilitate nerve regeneration. Future studies will be needed to define the role SCs and macrophages each or combined in these processes.

Autophagy is a conserved lysosomal degradation pathway that eliminates damaged organelles and pathological proteins to maintain intracellular homeostasis 17. Accumulating evidence suggests that myelin debris clearance is associated with autophagy 108, 109. Inhibition of autophagy by genetic knockout or a pharmacological approach resulted in delayed myelin fragmentation clearance in injured peripheral nerves 26, 110. Furthermore, enhancing autophagy may maintain microtubule stabilization, and promote peripheral nerve regeneration and functional recovery in the adult nervous system 24, 111. Autophagy is also regarded as a regulatory mechanism for structural plasticity of SCs during postnatal life 25. As such, we speculated that NGF promotion of myelin debris clearance is probably related to SC-medicated autophagic activation. This notion is supported by our results showing that NGF treatment enhanced the expression of autophagy-related proteins (including LC3, ATG-7, ATG-5 and Beclin-1) levels. Significantly, when autophagic inhibitor 3-MA blocked NGF-induced autophagic increase, myelin debris removal was retarded but never regeneration.

We further investigated the potential molecular mechanisms responsible for NGF regulation of SC autophagy during WD. AMPK and mTOR are two antagonistic regulators governing cellular autophagic dynamic homeostasis 51. Autophagy is promoted by AMPK signaling activation 112. Of particular note, AMPK coordinates with mTOR to regulate the homeostasis of autophagic-related neurodegenerative disorders 51, 113, 114. For example, metformin activates autophagy to exert a neuroprotective effect in spinal cord injury and focal cerebral ischemia via regulation of the AMPK/mTOR signaling pathway 91, 115. Our study has established that NGF acts on p75^NTR^ to regulate AMPK/mTOR to enhance SC autophagy. We found that NGF increased p75^NTR^ expression and AMPK phosphorylation. Moreover, suppressing p75^NTR^ or AMPK activation by pharmacological or genetic methods attenuated the NGF-induced increase in autophagy in SCs, which further resulted in a delay of myelin clearance and nerve regeneration.

Ideally, our findings and conclusions herein will be further validated using the SC-specific atg7 KO mice. A recent study using the SC conditional knockout atg7 mice has demonstrated that nerve injury induced a transient but robust activation of the mTOR pathway, that was necessary for proper myelin clearance 116. Interestingly, the authors have demonstrated that c-Jun activation in SCs is necessary for nerve injury and repair under their experimental settings 116. Our results that NGF suppressed mTOR activation after nerve injury appear to be inconsistent with these findings and conclusions. There are a number of possible explanations: we found that NGF increased the expression of autophagic proteins including LC3, ATG-7, ATG-5 and Beclin-1, it is not clear if loss of atg7 alone can be compensated by the increase of other proteins such LC3, ATG-5 and Beclin-1; 2) NGF binds to p75^NTR^ and activates small GTPases such as Rac 117 and CDC42 118 to increase c-Jun as well. The Rac/CDC42 pathways to increase c-Jun are independent from mTOR. Therefore, it is likely that administration of NGF following nerve injury, as in our current study, suppressed mTOR while boosting c-Jun level in SCs. Together, these molecular events regulate myelination and plasticity to facilitate myelin clearance and nerve regeneration.

In summary, our study has provided evidence supporting that NGF acts through the p75^NTR^/AMPK/mTOR signaling pathways to increase autophagy in SCs to facilitate myelin clearance and nerve repair. Importantly, this signaling cascade-induced autophagy activation is closely associated with clearing myelin fragments during WD, which ultimately contribute to the recovery process of injured peripheral nerves (Schematic 1). Therefore, the connection between NGF and autophagy in SCs will serve as a novel therapeutic strategy for nerve repair and regeneration.

## Supplementary Material

Supplementary figures.

## Figures and Tables

**Schematic 1:**
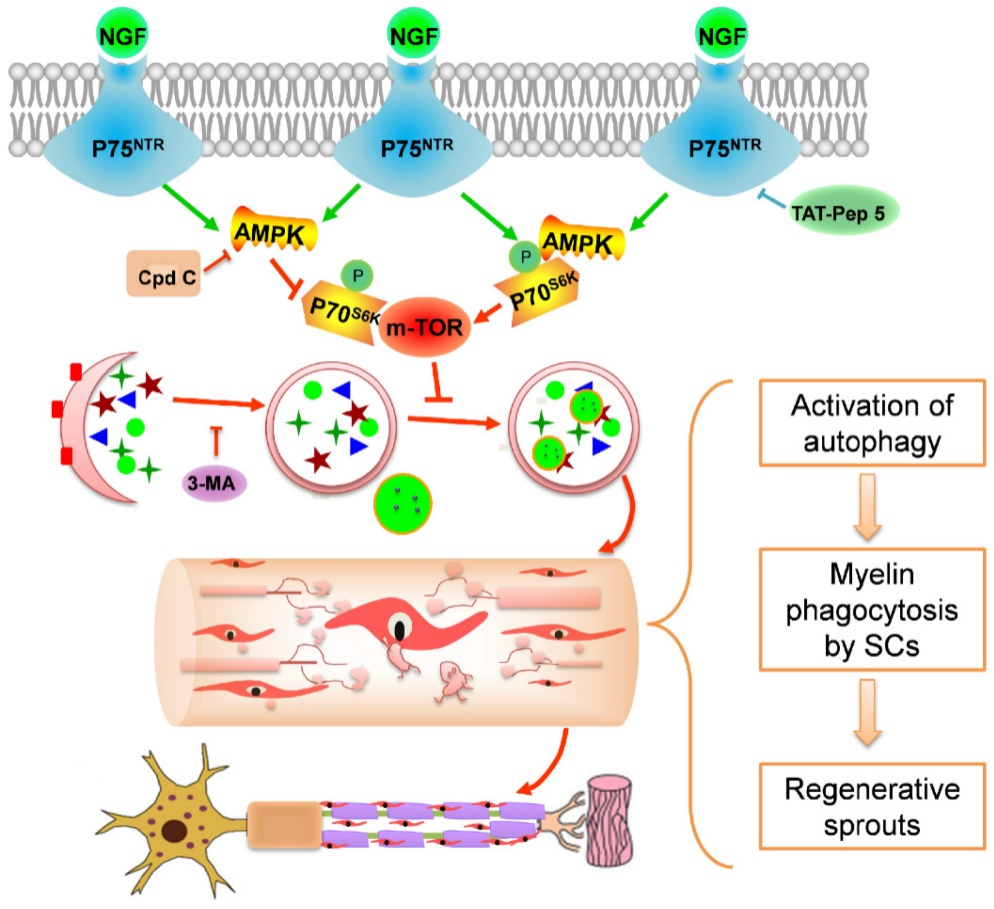
** Potential molecular mechanism by which NGF regulates myelin clearance and axon regeneration following PNI.** Exogenous NGF binds to the p75^NTR^ in SC to activate the AMPK/mTOR signing pathway to enhance autophagy and drive autophagic flux. This dynamic regulation process in SCs promotes engulfing and degradation of myelin fragments, thus shortening the time of myelin remodeling and axon growth for injured peripheral nerve.

**Table 1:** Primers used for RT-PCR in this study

Gene	Prime sequence	Product size (bp)	Serial number
β-actin	F: GCAAGTGCTTCTAGGCGGACTG R: CTGCTGTCACCTTCACCGTTCC	195	NM_001101683.1
MBP	F: AGTCCGACGAGCTACAGACCATC R: TACTTGGAGCCGTGCCTCTGG	106	XM_017338987.1
MPZ	F: TCATCGAGATGGAGCTACGGAAGG R: GGCGTTCTTGAGGCTGGTTCTG	89	XM_008264187.2
MAG	F: TGCCATCTTAATCGCCATCGTCTG R: CGCTTCTCGCTCTCATACTTCTCG	159	XM_017338174.1
MAP-2	F: GATCTGGCAGGCACAAGGTCAAG R: TTCCTCAACTACCGTCTCCGATGG	96	XM_017343068.1
GAP-43	F: GAAGGCGAGGCTGACCAAGAAC R: AGACGTGAGCAGGACAGGAAGG	141	XM_008266894.2

**Table 2:** Quantitative analysis of related proteins expression from immunoblotting in figure 8

	Mean ± SEM		Statistical analysis			
Protein	NGF	NGF+TAT-Pep 5	d.f.	t value	*P* value	Significant
p75^NGF^	1.00 ± 0.11	0.63 ± 0.07	4	4.657	0.019	*
*p*-AMPK/AMPK,	1.00 ± 0.10	0.66 ± 0.07	4	3.403	0.042	*
*p*-p70s6k/p70s6k	1.00 ± 0.08	1.57 ± 0.07	4	6.289	0.0081	**
*p*-mTOR/mTOR	1.00 ± 0.04	1.35 ± 0.08	4	4.992	0.038	*
ATG-7	1.00 ± 0.45	0.45 ± 0.20	4	4.657	0.043	*
ATG-5	1.00 ± 0.35	0.68 ± 0.31	4	3.595	0.036	*
Beclin-1	1.00 ± 0.28	0.60 ± 0.32	4	5.307	0.013	*
P62	1.00 ± 0.38	1.52 ± 0.40	4	4.159	0.025	*
LC3II/I	1.00 ± 0.26	0.78 ± 0.26	4	3.965	0.029	*
MBP	1.00 ± 0.33	1.30 ± 0.41	6	3.404	0.027	*
MPZ	1.00 ± 0.28	1.22 ± 0.30	4	4.385	0.022	*

The value of each protein expression was relative to the NGF group.^ *^P < 0.05, ^**^P < 0.01, compared with the NGF group.

**Table 3:** Quantitative analysis of related proteins expression from immunoblotting in figure 9

	Mean ± SEM			Statistical analysis		
Protein	PNI	NGF	NGF+Cpd C	F value	PNI vs. NGF	NGF vs. NGF+Cpd C
*p*-AMPK/AMPK,	1.00 ± 0.09	2.06 ± 0.23	1.10 ± 0.12	F_(2, 6)_ = 21.32	^**^P = 0.0052	^*^P = 0.019
*p*-p70s6k/p70s6k	1.00 ± 0.09	0.55 ± 0.03	0.92 ± 0.10	F_(2, 6)_ = 13.87	^**^P = 0.0041	^*^P = 0.047
*p*-mTOR/mTOR	1.00 ± 0.06	0.61 ± 0.04	0.99 ± 0.09	F_(2, 6)_ = 16.17	^**^P = 0.0063	^*^P = 0.045
ATG-7	1.00 ± 0.05	2.16 ± 0.26	1.40 ± 0.15	F_(2, 6)_ = 16.44	^**^P = 0.0051	^*^P = 0.042
ATG-5	1.00 ± 0.11	1.82 ± 0.18	1.27 ± 0.09	F_(2, 6)_ = 15.41	^**^P = 0.0073	^*^P = 0.038
Beclin-1	1.00 ± 0.13	1.66 ± 0.11	0.85 ± 0.12	F_(2, 6)_ = 19.51	^**^P = 0.0087	^**^P = 0.0084
P62	1.00 ± 0.08	0.51 ± 0.04	0.77 ± 0.09	F_(2, 6)_ = 17.29	^**^P = 0.0045	^*^P = 0.035
LC3II/I	1.00 ± 0.07	1.53 ± 0.11	0.96 ± 0.08	F_(2, 6)_ = 20.05	^**^P = 0.0076	^*^P = 0.014
MBP	1.00 ± 0.06	0.44 ± 0.05	0.82 ± 0.08	F_(2, 9)_ = 24.80	^**^P = 0.0012	^*^P = 0.010
MPZ	1.00 ± 0.09	0.61 ± 0.06	0.94 ± 0.07	F_(2, 6)_ = 11.20	^*^P = 0.038	^*^P = 0.026

The value of each protein expression was relative to the PNI group.^ *^P < 0.05, ^**^P < 0.01.

## Abbreviations

WD: Wallerian degeneration
PNS: peripheral nervous system
SCs: Schwann cells
SC: Schwann cell
PNI: peripheral nerve injury
NGF: Nerve growth factor
TrkA: tyrosine kinase receptor A
p75NTR: the 75kD p75 neurotrophin receptor
AMPK: AMP-activated protein kinase
mTOR: mammalian target of rapamycin
p70s6k: p70 ribosomal S6 kinase
Cpd C: Compound C
3-MA: 3-methyladenine
CQ: chloroquine
HE: Hematoxylin and eosin
DAPI: 4' 6-Diamidino-2-phenylindole-dihydrochlorid
OTC: cutting temperature compound
TEM: transmission electron microscopy
SEM: standard error of mean
LAMP1: lysosomal associated membrane protein 1
APs: autophagosomes
Masson: Masson's trichrome staining
LFB: Luxol fast blue
TB: toluidine blue
ORO: oil red O
IOD: the integrated option density
DRG: dorsal root ganglion
