# Supplementary material for: Nerve growth factor activates autophagy in Schwann cells to enhance myelin debris clearance and to expedite nerve regeneration
Source: Theranostics. 2020 Jan 1;10(4):1649–77. doi: 10.7150/thno.40919 (PMC6993217; doi:10.7150/thno.40919)
Supplement: Supplementary file 1 — Supplementary figures. [file thnov10p1649s1.pdf]

Supplemental Data

Nerve growth factor activates autophagy in Schwann cells to enhance myelin debris clearance and to expedite nerve regeneration

Rui Li [1, 2], Duohui Li [1], Chengbiao Wu [2], Libing Ye [1], Yanqing Wu [3], Yuan Yuan [1], Shengnan Yang [1], Ling Xie [1], Yuqin Mao [1], Ting Jiang [1], Yiyang Li [1], Jian Wang [1], Hongyu Zhang [1], Xiaokun Li [1], Jian Xiao [1*]

[1] Department of Peripheral Neurosurgery, The First Affiliated Hospital and School of Pharmaceutical Sciences, Wenzhou Medical University, Wenzhou, Zhejiang, 325000, China

[2] Research Center, Affiliated Xiangshang Hospital, Wenzhou Medical University, Ningbo, Zhejiang, 315700, China

[3] The Institute of Life Sciences, Wenzhou University, Wenzhou 325035, China

[*]Corresponding authors: Jian Xiao, PhD, xfxj2000@126.com

SUPPLEMENTAL METHODS

Nerve fiber explant culture

Sciatic nerve explant cultures were performed as reported by Park et al [1]. Briefly, sciatic nerves were extracted, and the connective tissues removed in calcium/magnesium-free Hank's buffered solution under a stereomicroscope. The sciatic nerves were then cut into small explants of 3-4 mm in length. The explants were then seeded in 6-well plates in Dulbecco's modified medium containing 10% fetal bovine serum (Gibco) at 37℃ with 5% CO2 for 2 h (DIV 0). NGF (Sigma–Aldrich, SRP3015, final concentration: 50ng/ml) with/without chloroquine (CQ, Aladdin, C193834, final concentration: 100 μM) was added to the culture media. The sciatic explants were stained for LC3 (red) and F-actin (green) at DIV 1, 3 and 5. F-actin was labeled with fluorescein phalloidin (Solarbio, CA1620, 1:1000).

Isolation and cultivation of primary DRG neurons

Dissociated DRG neurons were prepared from 2 month-old male Wistar male rats, as described previously by Vernon, C. G [2]. Briefly, adult rats were deeply anesthetized and the DRGs were freshly dissected. After stripping off surrounding fat and connective tissue as well as blood vessels, the DRG were transferred in 48- well plates coated poly-L-lysin. The DRG culture medium supplemented with 50 ng/mL NGF [3] plus GW441756 (a TrkA kinase inhibitor, 2 nM [4]) or TAT-Pep5 (the $p75^{NTR}$ inhibitor, 10 μM [5]). After culturing for 3 days, the neurite outgrowth was detected through chicken anti-NF-200 direct immunofluorescent staining.

Reference

1 Park BS, Kim HW, Rhyu IJ, Park C, Yeo SG, Huh Y, *et al*. Hydrogen sulfide is essential for Schwann cell responses to peripheral nerve injury. Journal of Neurochemistry. 2015; 132: 230–42.

2 Vernon CG, Swanson GT. Neto2 Assembles with Kainate Receptors in DRG Neurons during Development and Modulates Neurite Outgrowth in Adult Sensory Neurons. J Neurosci. 2017; 37: 3352-63.

3 Tanaka Y, Niwa S, Dong M, Farkhondeh A, Wang L, Zhou R, *et al*. The Molecular Motor KIF1A Transports the TrkA Neurotrophin Receptor and Is Essential for Sensory Neuron Survival and Function. Neuron. 2016; 90: 1215-29.

4 Ohta M, Chosa N, Kyakumoto S, Yokota S, Okubo N, Nemoto A, *et al*. IL1beta and TNFalpha suppress TGFbetapromoted NGF expression in periodontal ligamentderived fibroblasts through inactivation of TGFbetainduced Smad2/3 and p38 MAPKmediated signals. Int J Mol Med. 2018; 42: 1484-94.

5 Yamashita T, Tohyama M. The p75 receptor acts as a displacement factor that releases Rho from Rho-GDI. Nat Neurosci. 2003; 6: 461-7.

SUPPLEMENTAL FIGURE LEGENDS

Fig. S1 NGF facilitating DRG outgrowth through Trk A. **(A)** Confocal fluorescent images of DRGs, stained with NF-200 (green), the nuclei were labeled with DAPI. Scale bar = 500 μm. **(B, C)** Quantification of the number and length of axon in the DRG treating with NGF, TAT-Pep 5, and GW441756 alone or in combination. Data are represented as the means ± SEM; the experiment was repeated in triplicate. Axon length $F_{(2, 6)} = 107.70$, $P_{\text{NGF versus NGF+TAT-Pep-5}} = 0.256$ (n.s), ***$P_{\text{NGF versus NGF+GW441756}} < 0.001$; Axon number $F_{(2, 6)} = 62.46$, $P_{\text{NGF versus NGF+TAT-Pep-5}} =$ 0.442 (n.s), ***$P_{\text{NGF versus NGF+GW441756}} < 0.001$.

Fig. S2 NGF effect on nerve regeneration in PNI is mediated by $p75^{NTR}$. **(A, B)** Representative images of HE staining and immunostaining of NF-200 (green) and MBP (red) in PNI rats receiving NGF with/without K252a (Left) or TAT-Pep5 (Right) treatment. **(C-E)** Quantification of nerve fibers, NF-200 and MBP positive area in both groups. Data are represented as the means ± SEM; n = 3 rats per group. Nerve fibers: $P_{\text{NGF vs NGF+K252a}} = 0.571$ (n.s), t = 0.616, d.f. = 4; *$P_{\text{NGF vs NGF+TAT-Pep5}} = 0.016$, t = 5.576, d.f. = 4. NF-200: $P_{\text{NGF vs NGF+K252a}} = 0.478$ (n.s), t = 0.781, d.f. = 4; *$P_{\text{NGF vs NGF+TAT-Pep5}} = 0.027$, t = 4.377, d.f. = 4. MBP $P_{\text{NGF vs NGF+K252a}} = 0.178$ (n.s), t = 1.632, d.f. = 4; *$P_{\text{NGF vs NGF+TAT-Pep5}} = 0.024$, t = 6.604, d.f. = 4.

Fig. S3 TrkA was not involved in NGF-medicated autophagy enhancement, myelin clearance and nerve reestablishment after injury. **(A-D)** Representative western blotting and quantification data of *p*-AMPK, AMPK, *p*-p70s6k, p70s6k, *p*-mTOR and mTOR in NGF and NGF+GW441756 groups on day 5 after injury. Data are the mean values ± SEM; n = 3 independent experiments. *p*-AMPK/AMPK $P_{\text{NGF versus NGF+GW441756}} = 0.24$ (n.s), t = 1.470, d.f. = 4; *p*-p70s6k/p70s6k $P_{\text{NGF versus NGF+GW441756}} = 0.97$ (n.s), t = 0.043, d.f. = 4; *p*-mTOR/mTOR $P_{\text{NGF versus NGF+GW441756}} = 0.20$ (n.s), t = 1.854, d.f. = 4. **(E-I)** Western blotting was performed to detect ATG-7, ATG-5, Beclin-1 and LC3 levers in those two groups at 5 days post-crush. Data are the mean values ± SEM; n = 3 independent experiments. ATG-7 $P_{\text{NGF versus NGF+GW441756}} = 0.90$ (n.s), t = 0.141, d.f. = 4; ATG-5 $P_{\text{NGF versus NGF+GW441756}} = 0.94$ (n.s), t = 0.088, d.f. = 4; Beclin-1 P_{NGF}

$_{\text{versus NGF+GW441756}}$ = 0.66 (n.s), t = 0.490, d.f. = 4; LC3II/I $P_{\text{NGF versus NGF+GW441756}}$ = 0.68 (n.s), t = 0.452, d.f. = 4. **(J, L)** Immunostaining for MPZ (green) and GFAP (red) in the damaged sciatic nerve (5 days) of NGF and NGF+GW441756 rats. Data are the mean values ± SEM; n = 3 rats per group. MPZ $P_{\text{NGF versus NGF+GW441756}}$ = 0.86 (n.s), t = 0.192, d.f. = 4. **(K)** Representative TEM images and co-immunostaining for MBP (red)/NF-200 (green) of 14 day sections in each experimental group. **(M-P)** Quantification of the number of myelinated fibers, G-ratio, positive staining NF-200 and MBP areas in both groups from K. Data represents the means ± SEM; n = 3 rats per group. Myelinated fibers $P_{\text{NGF versus NGF+GW441756}}$ = 0.69 (n.s), t = 0.424, d.f. = 4; G-ratio $P_{\text{NGF versus NGF+GW441756}}$ = 0.99 (n.s), t = 0.017, d.f. = 4; MBP $P_{\text{NGF versus NGF+GW441756}}$ = 0.81 (n.s), t = 0.264, d.f. = 4; NF-200 $P_{\text{NGF versus NGF+GW441756}}$ = 0.78 (n.s), t = 0.304, d.f. = 4. Significance was determined with the unpaired t-test with Welch's correction.

Fig. S4 NGF promoted degradation of myelin-associated proteins after 5 days post-surgery. **(A)** Western blotting analysis of MBP and MPZ in the sham, PNI and PNI+NGF groups. GAPDH was served as a loading control. **(B, C)** The optical density of the MBP and MPZ proteins in each group was quantitated and presented as mean ± SEM; MBP $F_{(2, 21)} = 31.17$, $^{**}P_{\text{sham versus PNI}} = 0.0073$, $^{*}P_{\text{PNI versus PNI+NGF}} = 0.019$; MPZ $F_{(2, 21)} = 37.11$, $^{*}P_{\text{sham versus PNI}} = 0.036$, $^{**}P_{\text{PNI versus PNI+NGF}} = 0.0037$. n = 8 independent experiments.

Fig. S5 NGF enhanced myelin degradation and promoted axonal regeneration after injury. **(A)** Electron microscopic images of sections of lesioned nerves at 1, 3 and 5 days after crush injury. Control sciatic nerve was marker as 0 day. Scale bar = 5 μm. **(B, C)** Quantitative results of the numbers of newborn and abnormal myelin sheaths per 1000 μm^2 at different time points following injury. Data are presented as mean ± SEM; n = 3 rats per group. Abnormal myelin: $F_{(2, 6)} = 36.99$, $^{*}P_{\text{1d versus 3d}} = 0.024$, $^{**}P_{\text{3d versus 5d}} = 0.0063$; Newborn myelin: $F_{(2, 6)} = 31.20$, $^{*}P_{\text{1d versus 3d}} = 0.017$, $^{*}P_{\text{3d versus 5d}} = 0.027$. **(D)** Sciatic nerve explants were stained for F-actin (green) and LC3 (red). The explants were cultured for 0, 1, 3 and 5 days in the presence of NGF with/without CQ. Scale bar = 10 μm. **(E, F)** Quantification of fluorescence intensity of F-actin and LC3 pixels from (D). Data are presented as mean ± SEM. n = 3 independent experiments. Significance was determined with the unpaired t-test with Welch's correction. F-actin $^{**}P_{\text{NGF-5d}}$

$_{\text{versus NGF+CQ-5d}}$ = 0.0085, $^{***}P_{\text{5d-NGF versus 0d-NGF}} < 0.001$, $P_{\text{NGF-0d versus NGF+CQ-0d}} = 0.87$ (n.s); LC3 $^{***}P_{\text{NGF-5d versus NGF+CQ-5d}} < 0.001$, $^{**}P_{\text{NGF-3d versus NGF+CQ-3d}} = 0.0045$, $^{*}P_{\text{NGF-1d versus NGF+CQ-1d}} = 0.017$, $P_{\text{NGF-0d versus NGF+CQ-0d}} = 0.024$ (n.s), $^{***}P_{\text{5d-NGF versus 0d-NGF}} < 0.001$.

Fig. S6 Macrophages play a role in myelin removal after PNI. **(A)** Immunofluorescence staining against CD68-positive macrophages from PNI rats receiving either SiO_2 or Cyclosporin with and without NGF at 5 days post-injury. **(B)** ORO staining was performed on longitudinal nerve sections 5 days post-surgery in each group. **(C)** Immunostaining for MPZ (green) showing residual myelin fragments on day 5 after injury. **(D-F)** Statistical analysis of macrophage numbers, ORO positive area and myelin accumulation per 100 μm^2 in each group from (A), (B) and (C). Data are presented as mean ± SEM. n = 3 rats per group. Significance was determined with the unpaired t-test with Welch's correction. Macrophage numbers $P_{\text{PNI vs PNI+NGF}} = 0.42$ (n.s), $^{**}P_{\text{NGF vs NGF+SiO2}} = 0.0054$, $^{**}P_{\text{NGF vs NGF+Cyclosporin A}} = 0.0052$, $P_{\text{NGF+SiO2 vs NGF+Cyclosporin A}} = 0.54$ (n.s), $^{**}P_{\text{PNI vs PNI+SiO2}} = 0.0043$, $^{**}P_{\text{PNI vs PNI+Cyclosporin A}} = 0.0045$, $P_{\text{PNI+SiO2 vs PNI+Cyclosporin A}} = 0.57$ (n.s); ORO positive area $^{**}P_{\text{PNI vs PNI+NGF}} = 0.0041$, $P_{\text{NGF vs NGF+SiO2}} = 0.72$ (n.s), $P_{\text{NGF vs NGF+Cyclosporin A}} = 0.89$ (n.s), $P_{\text{PNI vs PNI+SiO2}} = 0.19$ (n.s), $P_{\text{PNI vs PNI+Cyclosporin A}} = 0.23$ (n.s); MPZ positive area $^{**}P_{\text{PNI vs PNI+NGF}} = 0.0061$, $P_{\text{NGF vs NGF+SiO2}} = 0.31$ (n.s), $P_{\text{NGF vs NGF+Cyclosporin A}} = 0.59$ (n.s), $P_{\text{PNI vs PNI+SiO2}} = 0.32$ (n.s), $P_{\text{PNI vs PNI+Cyclosporin A}} = 0.65$ (n.s).

Fig. S7 Suppressing macrophages activation has no significant impact on NGF-mediated peripheral nerve repair. **(A)** Immunohistochemical staining for macrophage marker CD68 from PNI rats receiving SiO_2 or Cyclosprin A with and without NGF at 14 days post-injury. **(B)** Immunofluorescent staining against NF-200 (green) and MBP (red) in the longitudinal nerve sections at 14 days postsurgery. Nucleus were labeled with DAPI. **(C)** Representative transverse sections of crush injured sciatic nerve were stained with toluidine blue (TB) at 14 days postoperatively. **(D-G)** Quantification of macrophage numbers, NF-200 and MBP positive area, and myelin numbers per 100 μm^2 in each group from (A), (B) and (C). Data are presented as mean ± SEM.

Significance was determined with the unpaired t-test with Welch's correction. n = 3 rats per group. Macrophage numbers $P_{\text{PNI vs PNI+NGF}} = 0.62$ (n.s), $P_{\text{NGF vs NGF+SiO2}} = 0.67$ (n.s), $P_{\text{NGF vs NGF+Cyclosporin A}} = 0.80$ (n.s), $P_{\text{PNI vs PNI+SiO2}} = 0.65$ (n.s), $P_{\text{PNI vs PNI+Cyclosporin A}} = 0.76$ (n.s); NF-200 $^{**}P_{\text{PNI vs PNI+NGF}} = 0.0075$, $P_{\text{NGF vs NGF+SiO2}} = 0.91$ (n.s), $P_{\text{NGF vs NGF+Cyclosporin A}} = 0.92$ (n.s), $^{*}P_{\text{PNI vs PNI+SiO2}} = 0.047$, $^{*}P_{\text{PNI vs PNI+Cyclosporin A}} = 0.027$; MBP $^{**}P_{\text{PNI vs PNI+NGF}} = 0.0086$, $P_{\text{NGF vs NGF+SiO2}} = 0.46$ (n.s), $P_{\text{NGF vs NGF+Cyclosporin A}} = 0.33$ (n.s), $^{*}P_{\text{PNI vs PNI+SiO2}} = 0.043$, $^{*}P_{\text{PNI vs PNI+Cyclosporin A}} = 0.041$; myelin numbers $^{*}P_{\text{PNI vs PNI+NGF}} = 0.043$, $P_{\text{NGF vs NGF+SiO2}} = 0.93$ (n.s), $P_{\text{NGF vs NGF+Cyclosprin A}} = 0.84$ (n.s), $^{**}P_{\text{PNI vs PNI+SiO2}} = 0.0089$, $^{**}P_{\text{PNI vs PNI+Cyclosporin A}} = 0.0070$.

Fig. S8 Schematic showing the process of extraction and manipulation of collected sciatic nerve samples for preparing different staining. the preparation of tissue section for HE, LFB and TB staining was shown in **(a)**; the preparation of tissue section for immunofluorescence and ORO staining was shown in **(b)**; the preparation of tissue section for TEM was shown in **(c)**.

Figure S1

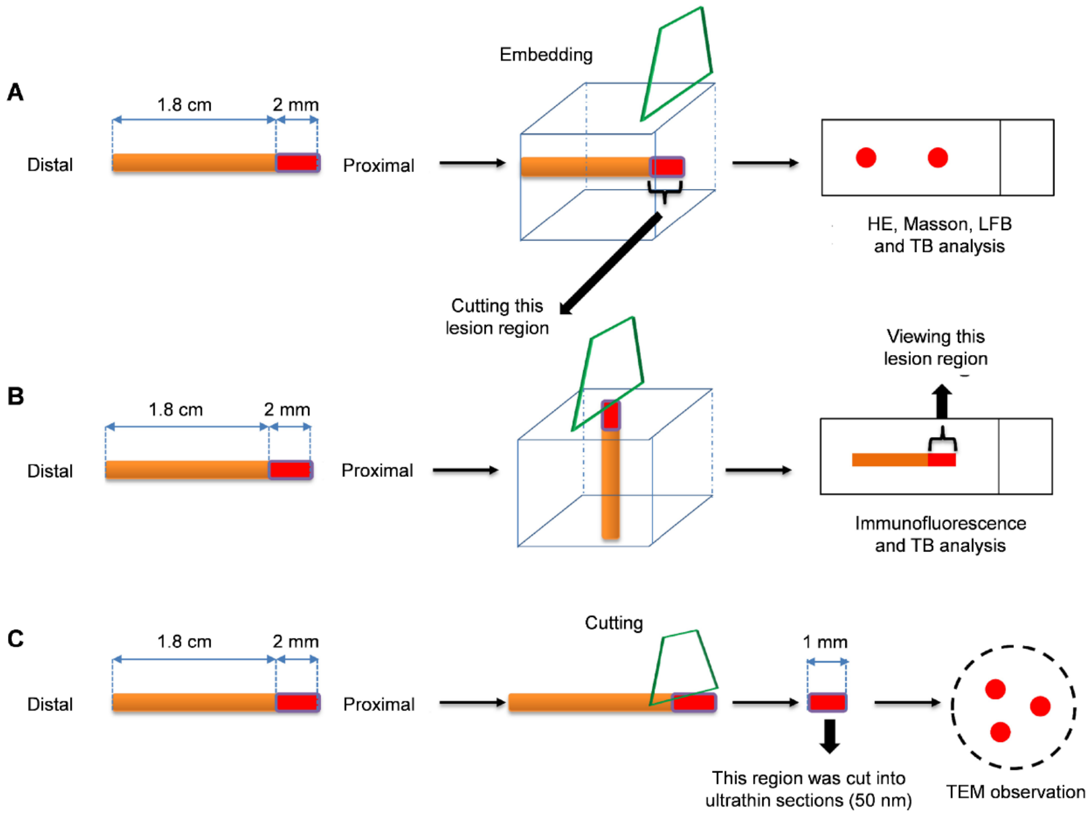
A
1.8 cm
2 mm
Distal
Proximal
Embedding
HE, Masson, LFB
and TB analysis
Cutting this
lesion region
B
1.8 cm
2 mm
Distal
Proximal
Viewing this
lesion region
Immunofluorescence
and TB analysis
C
1.8 cm
2 mm
Distal
Proximal
Cutting
1 mm
This region was cut into
ultrathin sections (50 nm)
TEM observation

Figure S2

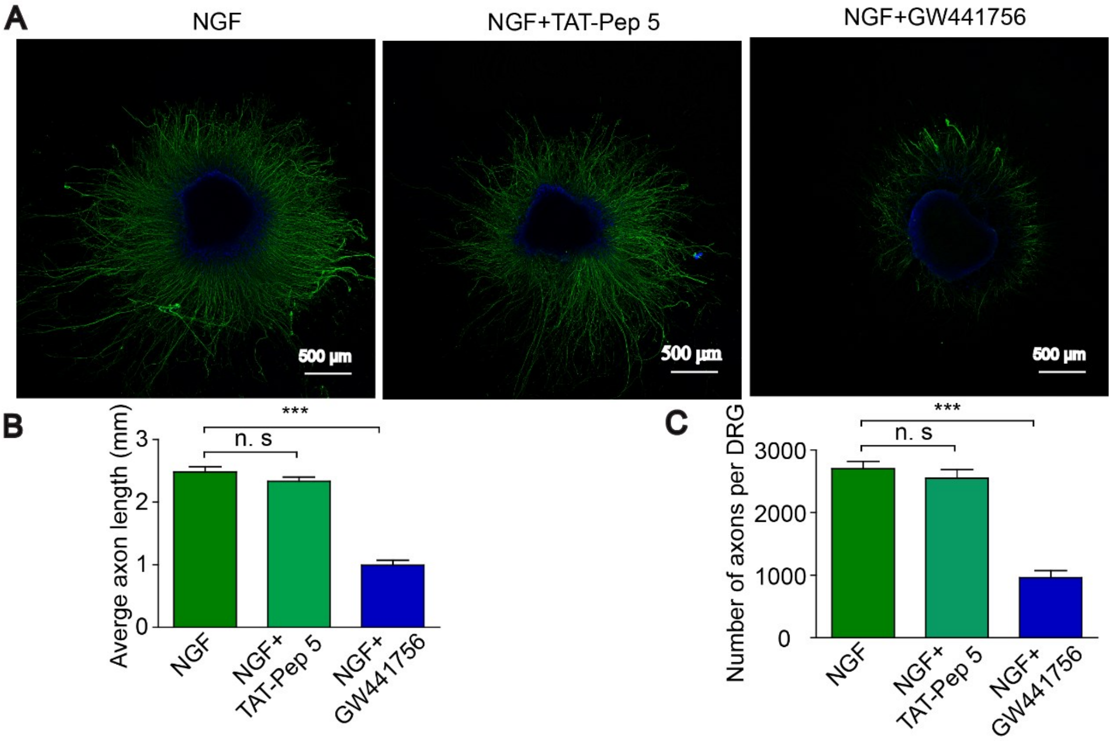
A
NGF
NGF+TAT-Pep 5
NGF+GW441756
500 μm
500 μm
500 μm
B
Averge axon length (mm)
n. s
***
3
2
1
0
NGF
NGF+ TAT-Pep 5
NGF+ GW441756
C
Number of axons per DRG
n. s
***
3000
2000
1000
0
NGF
NGF+ TAT-Pep 5
NGF+ GW441756

Figure S3

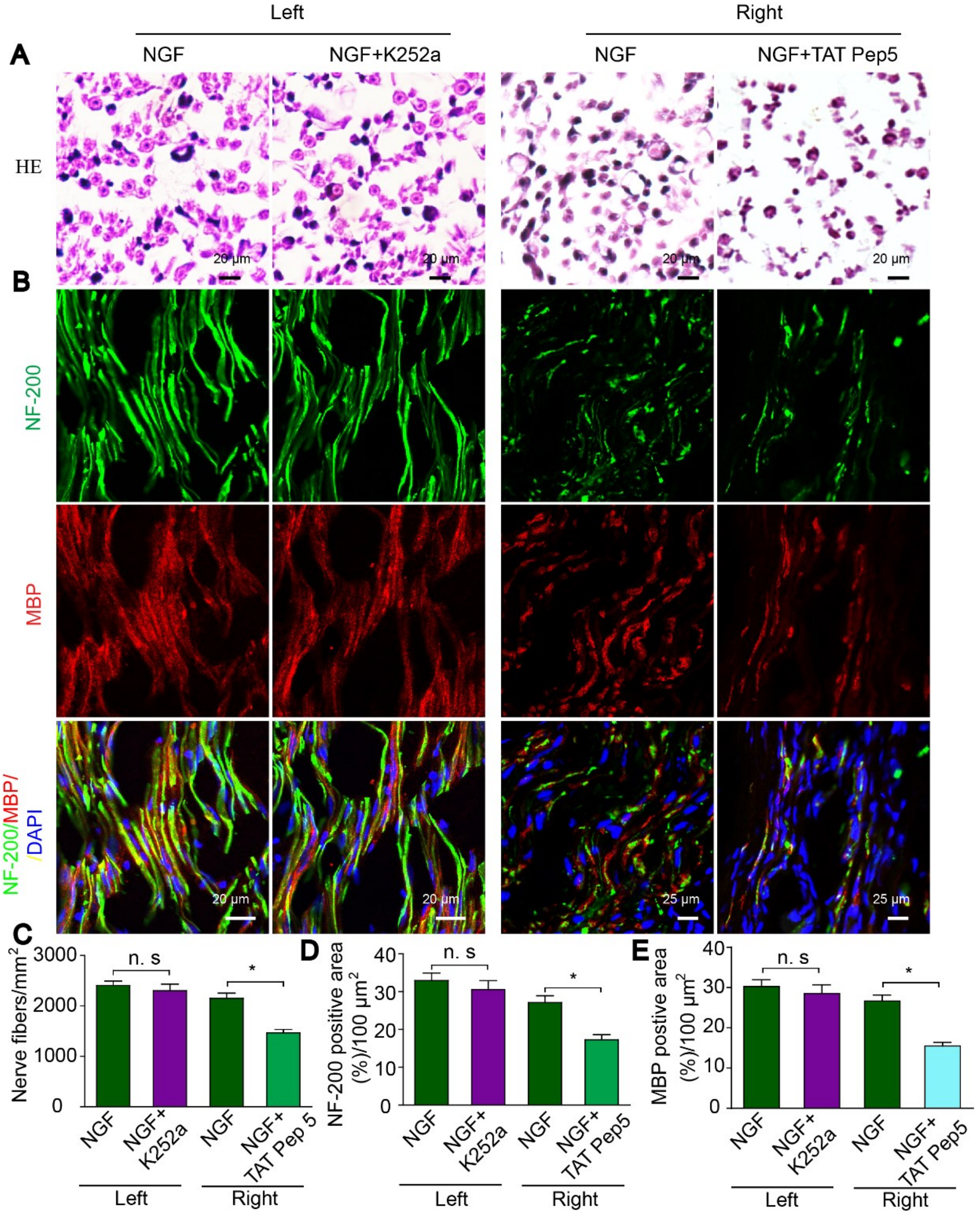

A
B
C
D
E
Left
Right
NGF
NGF+K252a
NGF
NGF+TAT Pep5
HE
NF-200
MBP
NF-200/MBP/DAPI
20 μm
25 μm
Nerve fibers/mm2
NF-200 positive area (%)/100 μm2
MBP postive area (%)/100 μm2
n. s
*
NGF+ K252a
NGF+ TAT Pep 5
NGF+ TAT Pep5

Figure S4

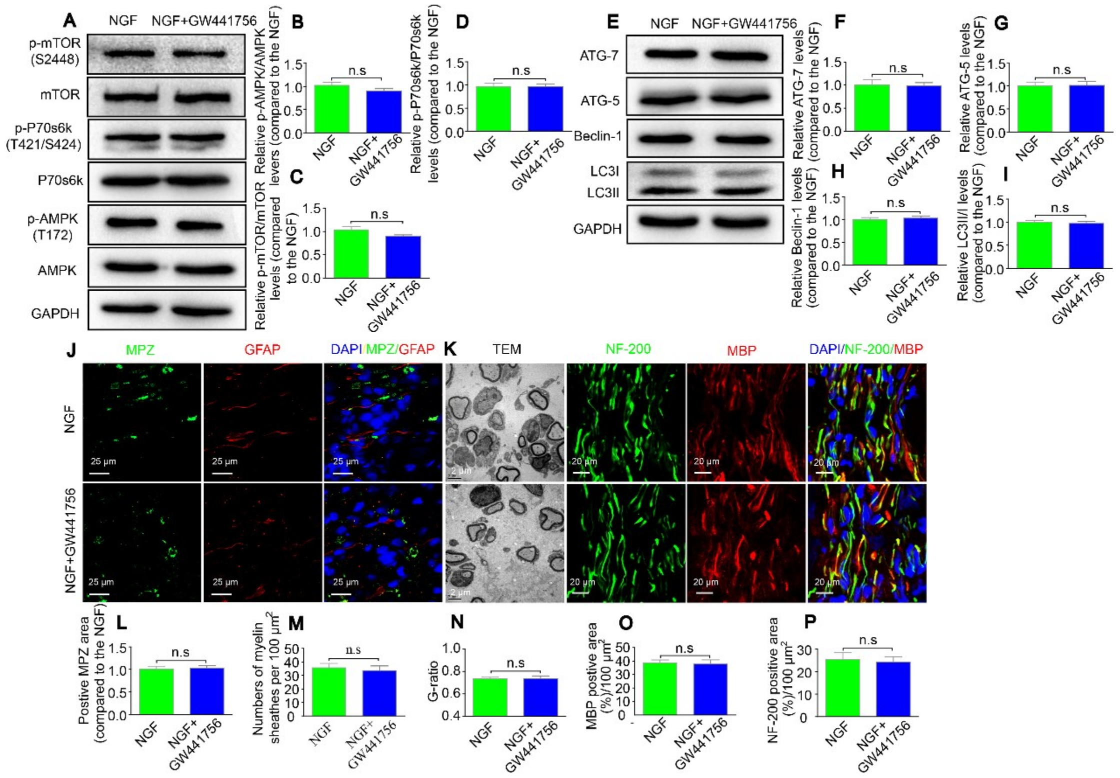

A
NGF
NGF+GW441756
p-mTOR (S2448)
mTOR
p-P70s6k (T421/S424)
P70s6k
p-AMPK (T172)
AMPK
GAPDH
B
Relative p-AMPK/AMPK levers (compared to the NGF)
n.s
NGF
NGF+ GW441756
C
Relative p-mTOR/mTOR levels (compared to the NGF)
D
Relative p-P70s6k/P70s6k levels (compared to the NGF)
E
ATG-7
ATG-5
Beclin-1
LC3I
LC3II
F
Relative ATG-7 levels (compared to the NGF)
G
Relative ATG-5 levels (compared to the NGF)
H
Relative Beclin-1 levels (compared to the NGF)
I
Relative LC3II/I levels (compared to the NGF)
J
MPZ
GFAP
DAPI/MPZ/GFAP
25 μm
K
TEM
NF-200
MBP
DAPI/NF-200/MBP
20 μm
2 μm
L
Postive MPZ area (compared to the NGF)
M
Numbers of myelin sheathes per 100 μm²
N
G-ratio
O
MBP postive area (%)/100 μm²
P
NF-200 positive area (%)/100 μm²

Figure S5

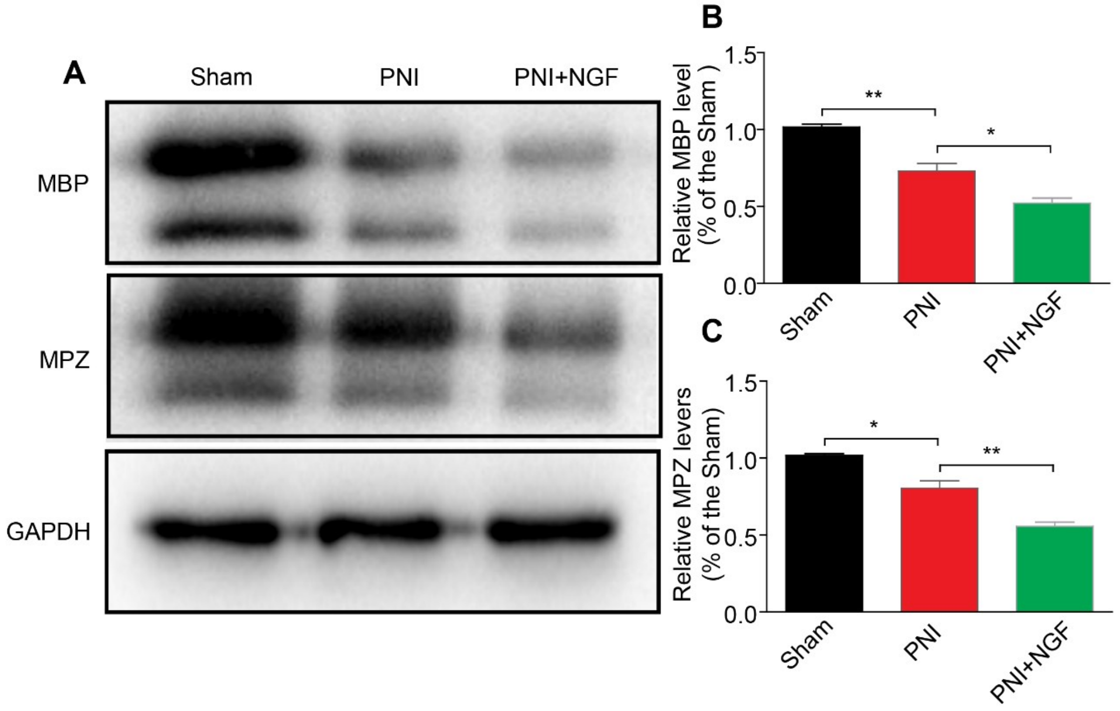

A
Sham
PNI
PNI+NGF
MBP
MPZ
GAPDH
B
Relative MBP level
(% of the Sham)
1.5
1.0
0.5
0.0
**
*
Sham
PNI
PNI+NGF
C
Relative MPZ levers
(% of the Sham)
1.5
1.0
0.5
0.0
*
**
Sham
PNI
PNI+NGF

Figure S6

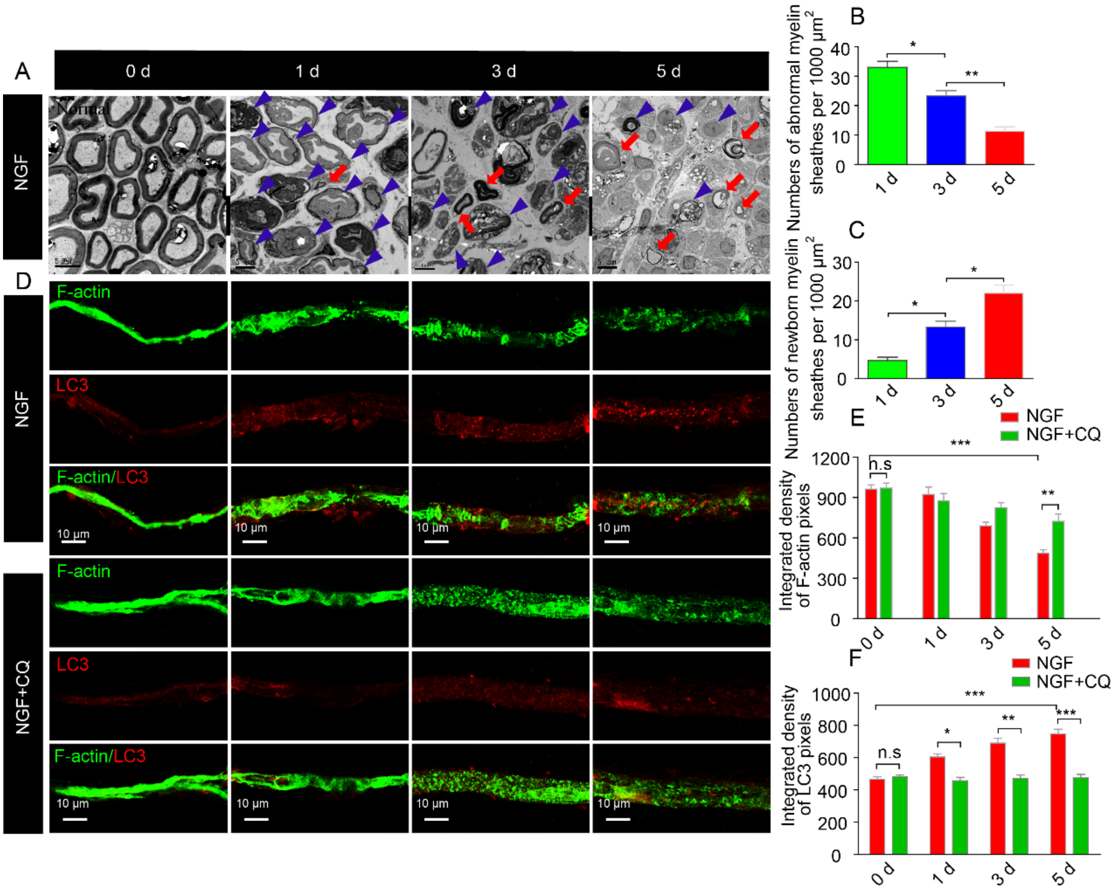
A
0 d
1 d
3 d
5 d
NGF
D
F-actin
LC3
F-actin/LC3
10 μm
NGF+CQ
B
Numbers of abnormal myelin sheathes per 1000 μm²
C
Numbers of newborn myelin sheathes per 1000 μm²
E
NGF
NGF+CQ
Integrated density of F-actin pixels
n.s
F
Integrated density of LC3 pixels

Figure S7

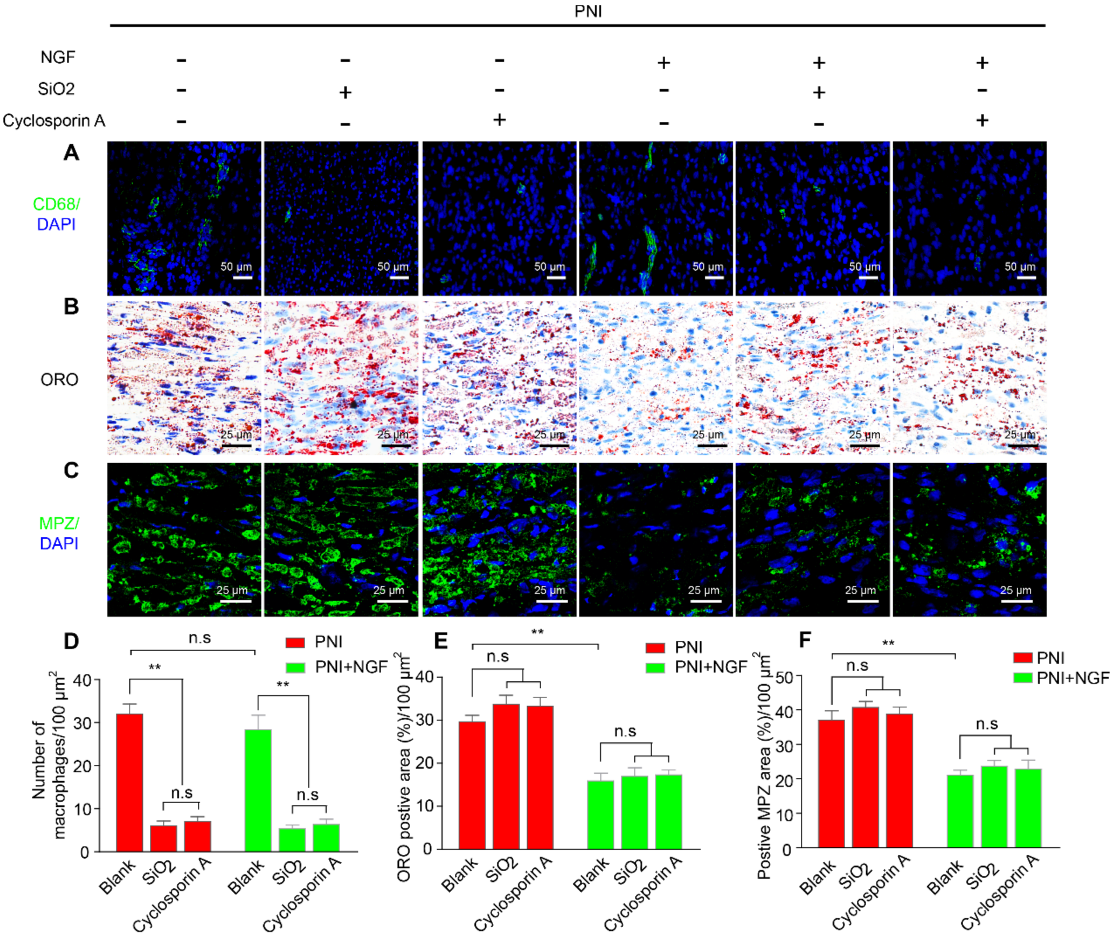

PNI
NGF
SiO2
Cyclosporin A
A
CD68/
DAPI
50 μm
B
ORO
25 μm
C
MPZ/
DAPI
25 μm
D
n.s
**
PNI
PNI+NGF
Number of macrophages/100 μm2
Blank
SiO2
Cyclosporin A
E
ORO postive area (%)/100 μm2
F
Postive MPZ area (%)/100 μm2

Figure S8

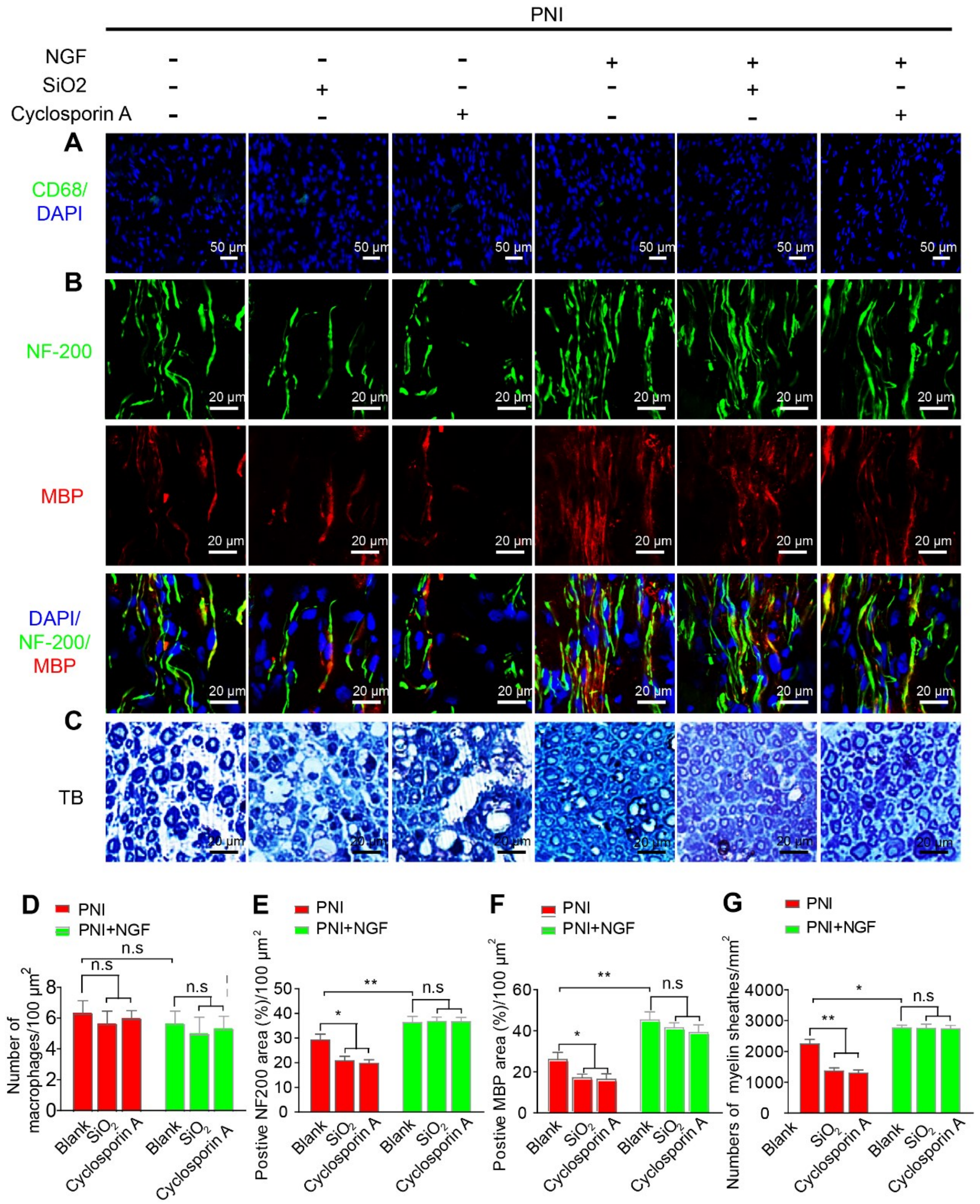
PNI
NGF
SiO2
Cyclosporin A
A
CD68/
DAPI
50 μm
B
NF-200
MBP
DAPI/
NF-200/
MBP
20 μm
C
TB
D
PNI
PNI+NGF
Number of macrophages/100 μm2
n.s
Blank
SiO2
Cyclosporin A
E
Postive NF200 area (%)/100 μm2
F
Postive MBP area (%)/100 μm2
G
Numbers of myelin sheathes/mm2
